# Different effects of social cognitive skills training in schizophrenia and autism spectrum disorder: A rater‐blinded, multicenter, randomized controlled trial

**DOI:** 10.1111/pcn.70106

**Published:** 2026-07-22

**Authors:** Sadao Otsuka, Satsuki Oe, Sumie Yamada, Haruka Kumoi, Ryoichi Suwabe, Tatsuro Iwane, Kosuke Tsurumi, Toshiya Murai

**Affiliations:** ^1^ Department of Psychiatry, Graduate School of Medicine Kyoto University Kyoto Japan; ^2^ Department of Clinical Psychology, Graduate School of Education Hyogo University of Teacher Education Kobe Japan; ^3^ Department of Health Promotion and Human Behavior, Graduate School of Medicine/School of Public Health Kyoto University Kyoto Japan; ^4^ Marui Mental Clinic Kyoto Japan; ^5^ Kyoto Prefectural Rakunan Hospital Uji Japan; ^6^ Department of Science for Well‐being and Security, Health Security Center Graduate School of Medicine, Kyoto University Kyoto Japan

**Keywords:** facial emotion recognition, mentalizing, reading the mind in the eyes test, social cognition training, social functioning

## Abstract

**Aim:**

Schizophrenia (SCZ) and autism spectrum disorder (ASD) are both characterized by social cognitive impairments that strongly predict poor functional outcomes. However, evidence‐based social cognitive interventions have been developed only for SCZ. This study investigated the feasibility and efficacy of social cognitive skills training (SCST), focusing on both common and different effects on SCZ and ASD.

**Methods:**

Forty‐seven adults with SCZ and/or ASD were randomly assigned to SCST (*n* = 24) or a treatment‐as‐usual control (CON; *n* = 23). The Japanese version of SCST developed for this study comprised weekly 2‐h group sessions over 6 weeks and targeted two social cognition levels: lower‐level emotional processing and higher‐level theory of mind. Blinded raters assessed social cognition, neurocognition, clinical symptoms, and functioning at baseline, post‐intervention, and 6‐week follow‐up.

**Results:**

SCST significantly improved facial emotion perception across diagnoses and demonstrated a trend‐level ASD‐specific improvement in theory of mind. These effects were maintained and generalized to improved global functioning only for ASD at follow‐up. High attendance and positive participant feedback in both groups indicated the intervention's feasibility.

**Conclusion:**

This study provides initial evidence supporting the feasibility and efficacy of social cognitive interventions as a transdiagnostic approach for SCZ and ASD and is the first to show functional benefits in adults with ASD. The findings highlight both shared and disorder‐specific mechanisms of social cognitive impairments and support the development of hybrid interventions that integrate transdiagnostic core components with tailored, diagnosis‐sensitive modules for diverse psychiatric populations.

**Clinical Trial Registration:**

The study protocol was preregistered on the University Hospital Medical Information Network Clinical Trials Registry (UMIN000041619; https://center6.umin.ac.jp/cgi‐open‐bin/ctr/ctr_view.cgi?recptno=R000047105; September 7, 2020).

Schizophrenia (SCZ) and autism spectrum disorder (ASD) are conceptualized as distinct disorders with unique symptoms and developmental onsets.^1^ However, the two disorders share some cognitive, neurobiological, and genetic features, including characteristics related to social cognition.^2^ Social cognition encompasses the psychological processes involved in perceiving and interpreting social information and that guide social behavior.^3^ These processes can be broadly categorized into lower‐level functions, such as emotion perception, and higher‐level functions, such as theory of mind.^4^ The neural systems that underlie these functions are partly different.^5^ Pioneering studies have demonstrated that higher and lower levels of social cognition are similarly impaired in both individuals with SCZ and adults with ASD without intellectual disability.^6^, ^7^, ^8^, ^9^ Furthermore, the severity of social cognitive impairments strongly predicts poor functional outcomes in both SCZ^10^, ^11^ and ASD.^12^, ^13^ Consequently, social cognition is recognized as a key treatment target, with improvement expected to enhance real‐world functioning in adults with these disorders.

In the comprehensive care of individuals with SCZ, broader and multifaceted psychosocial treatments have played an important role in promoting functional recovery.^14^ Among these, psychological interventions that specifically target social cognition, collectively known as social cognition training, emerged over the last two decades and appear promising.^15^, ^16^ A recent meta‐analysis of randomized controlled trials (RCTs) demonstrated that broad‐based programs addressing several social cognitive domains, including both lower‐ and higher‐level abilities, improved not only social cognition but also social functioning.^17^ In contrast, evidence for functional gains from single‐domain interventions has been less consistent. Additionally, group‐based interventions were more effective than individual interventions in improving social functioning.^17^ This evidence suggests that group programs targeting both social cognition levels are particularly promising. However, of the 46 RCTs included in that meta‐analysis, only five conducted follow‐up assessments at least 1 month post‐intervention, and in those cases, effect sizes tended to reduce over time. Thus, current challenges in developing social cognition training interventions for SCZ are to strengthen the durability of their effects and to determine the extent to which improvements in social cognition lead to improvements in social functioning. Additionally, the efficacy of social cognition training for Japanese individuals with SCZ remains unclear. Replication of these findings in Japan would support the cross‐cultural applicability of social cognitive interventions.

Psychosocial interventions, including those targeting social cognition, for adults with ASD remain limited.^18^ The number of adults diagnosed with ASD has increased markedly over the last two decades,^19^ and most individuals with ASD are not diagnosed until adulthood.^20^ Therefore, developing effective psychosocial treatments for these individuals, particularly those without intellectual disability, is an urgent clinical priority. Although social dysfunctions, including social cognitive impairments, in ASD and SCZ are considered to reflect the different mechanisms or developmental trajectories,^21^, ^22^ both disorders show overlapping difficulties in lower‐ and higher‐level social cognition. This makes it logical and efficient to adapt existing SCZ‐based social cognition training programs for adults with ASD. Among the broad‐based group interventions that have shown functional benefits for SCZ, social cognitive skills training (SCST)^23^, ^24^, ^25^ is particularly promising. A 6‐week short SCST program^26^ excludes sessions on attributional bias, for which evidence in ASD is limited, and instead focuses exclusively on emotion perception (lower‐level) and theory of mind (higher‐level), both of which are core difficulties in ASD. Therefore, SCST offers a strong conceptual framework for testing whether social cognition training is effective in adults with ASD. Notably, to date, no RCTs have examined whether psychological interventions specifically targeting social cognition can improve functional outcomes in this population. Therefore, rigorous investigation of this issue is warranted.

In the present study, we developed a Japanese version of SCST and conducted an RCT to evaluate its feasibility and efficacy in adults with SCZ and/or ASD. First, we examined whether the established effects of SCST on improving social cognition could be replicated in Japanese individuals with SCZ. Although the 6‐week short SCST program has shown benefits for social cognition, its generalization to functional outcomes and the durability of its effects have never been tested. To address this, our Japanese SCST incorporated unique homework assignments that promoted daily application of learned skills, and this study included a 6‐week follow‐up assessment. Second, we investigated whether SCST would produce similar benefits for adults with ASD. The effectiveness of SCST for both SCZ and ASD would support its validity as a transdiagnostic intervention. Conversely, disorder‐specific differences in treatment response could elucidate the distinct nature of social cognitive impairments in SCZ and ASD, informing the development of tailored interventions for each disorder. On the basis of previous SCST trials,^23^, ^24^, ^25^, ^26^ we hypothesized that (1) SCST would improve social cognition (primary outcome), particularly facial emotion perception, in both SCZ and ASD; (2) these improvements would be maintained at follow‐up and (3) generalize to functional improvements post‐intervention; and (4) SCST would have differing effects on SCZ and ASD.

## Methods

### Participants

Forty‐seven individuals with SCZ and/or ASD (26 men, 21 women) aged 20–65 years were recruited from outpatient psychiatric rehabilitation facilities at Kyoto University Hospital and two affiliated hospitals. According to the Diagnostic and Statistical Manual of Mental Disorders, Fifth Edition (American Psychiatric Association, 2013), 21 participants met the criteria for SCZ only, 23 participants for ASD only, and 3 participants for both SCZ and ASD. At the study design stage, eligibility criteria included schizophrenia spectrum disorder (SSD), encompassing SCZ and related psychotic disorders, to reflect the transdiagnostic nature of social cognitive impairments across the SCZ spectrum.^27^ However, all participants meeting SSD criteria in the present sample were diagnosed with SCZ; therefore, the manuscript primarily refers to this group as SCZ. Additionally, although participants aged ≥16 years were eligible, no individuals aged <20 years were enrolled. Exclusion criteria included a history of traumatic brain injury, neurological illness, substance/alcohol abuse, and current high risk for violence, suicide attempts, or severe impulsive behavior. Premorbid full‐scale intelligence quotient (IQ) was estimated as >80 for all participants, based on the Japanese Adult Reading Test (JART).^28^ Current full‐scale IQ was estimated using four subtests (Digit Symbol Coding, Matrix Reasoning, Digit Span, and Information) from the Japanese version of the Wechsler Adult Intelligence Scale, Third Edition (WAIS‐III),^29^, ^30^ following the recommendation of the Japanese WAIS‐III Publication Committee.^31^ Table 1 shows demographic, clinical, and intellectual characteristics. Given the earlier developmental onset of ASD, Table S1 reports participant characteristics according to diagnosis, with participants with both SCZ and ASD included in the ASD category. The required sample size to detect a between‐group difference in change was estimated as 52 participants, assuming a large effect based on prior trials of SCST for SCZ,^24^, ^25^ with α = 0.05 and power = 0.80. Due to the limited funding period and recruitment constraints, 47 participants were ultimately enrolled. The initially planned sample size of 88 participants was reduced during the recruitment phase due to slow enrollment. Participants were recruited between October 2020 and February 2022, and follow‐up continued until May 2022.

**Table 1:** Demographic and clinical characteristics of participants.

	SCST group, mean (SD)	*n*	CON group, mean (SD)	*n*	
Age (years)	43.21 (10.61)	24	38.00 (12.38)	23	*t*(45) = 1.55, *P* = 0.128
Sex (Male %)	54.17	24	56.52	23	*χ* ^2^(1) = 0.03, *P* = 0.871
Education (years)	14.50 (2.45)	24	13.83 (2.15)	23	*t*(45) = 1.00, *P* = 0.322
Diagnosis	SCZ: *n* = 10 ASD: *n* = 12 SCZ and ASD: *n* = 2	24	SCZ: *n* = 11 ASD: *n* = 11 SCZ and ASD: *n* = 1	23	
Duration of SCZ (years)	20.33 (10.23)	12	20.00 (9.64)	12	*t*(22) = 0.08, *P* = 0.935
Dosage (mg/day, RPD eq.)	3.87 (3.23)	19	3.33 (2.82)	18	*t*(35) = 0.52, *P* = 0.713
PANSS positive	15.17 (3.19)	12	13.50 (3.63)	12	*t*(22) = 1.20, *P* = 0.245
PANSS negative	17.83 (4.63)	12	17.00 (4.02)	12	*t*(22) = 0.47, *P* = 0.643
CARS2‐HF	35.86 (4.74)	14	33.42 (2.57)	12	*t*(24) = 1.59, *P* = 0.125
SPQ	40.63 (16.14)	24	35.35 (15.38)	23	*t*(45) = 1.15, *P* = 0.258
AQ	30.04 (6.53)	24	28.87 (7.88)	23	*t*(45) = 0.56, *P* = 0.581
JART‐estimated‐IQ	109.33 (8.79)	24	107.39 (8.10)	23	*t*(45) = 0.79, *P* = 0.435
WAIS‐III‐estimated‐IQ	97.25 (23.53)	24	94.17 (16.00)	23	*t*(45) = 0.52, *P* = 0.604
Rehabilitation time (h/w)	6.16 (4.68)	24	4.56 (4.34)	23	*t*(45) = 1.21, *P* = 0.231

AQ, autism spectrum quotient; ASD, autism spectrum disorder; CARS2‐HF, childhood autism rating scale, second edition, high‐functioning version; CON, control; IQ, intelligence quotient; JART, Japanese adult reading test; PANSS, positive and negative syndrome scale; RPD eq., risperidone equivalence; SCST, social cognitive skills training; SCZ, schizophrenia; SD, standard deviation; SPQ, schizotypal personality questionnaire; WAIS‐III, Wechsler adult intelligence scale, third edition.

### Study design and randomization

This study employed a parallel‐group, randomized, exploratory trial design with a 1:1 allocation ratio. No interim analyses or stopping guidelines were planned for this trial. Randomization was performed using a computer‐generated permuted block design with variable block sizes, stratified by intervention site, diagnosis (SCZ or ASD), and estimated IQ (SCZ: ≥90 vs <90; ASD: ≥100 vs <100). The allocation sequence was stored on a computer accessible only to the second author, and the personnel who enrolled and assigned participants had no access to the sequence.

### Procedures

After baseline assessment, participants in each diagnostic group were randomly assigned to either the SCST group (*n* = 24) or the treatment‐as‐usual control group (CON; *n* = 23). Baseline demographic, clinical, and cognitive characteristics were comparable between groups (see Table 1), indicating successful randomization. A flowchart of participant progress through the study is presented in Figure 1. Blinded raters conducted assessments of social cognition, neurocognition, clinical symptoms, and functioning at baseline (0 weeks), immediately post‐intervention (6 weeks), and 6 weeks post‐intervention (follow‐up at 12 weeks). Forty‐seven participants were randomized, 41 completed the post‐intervention assessment, and 40 completed the 6‐week follow‐up assessment. A 1‐year (52‐week) follow‐up assessment of functioning was also conducted; however, these long‐term follow‐up data were intended for analyses beyond the scope of the present manuscript and will therefore be reported separately. All assessments were administered individually by trained clinical psychologists. Participants received financial compensation for assessment time (¥1000/h). Data were collected between October 2020 and May 2022.

**Fig. 1:**
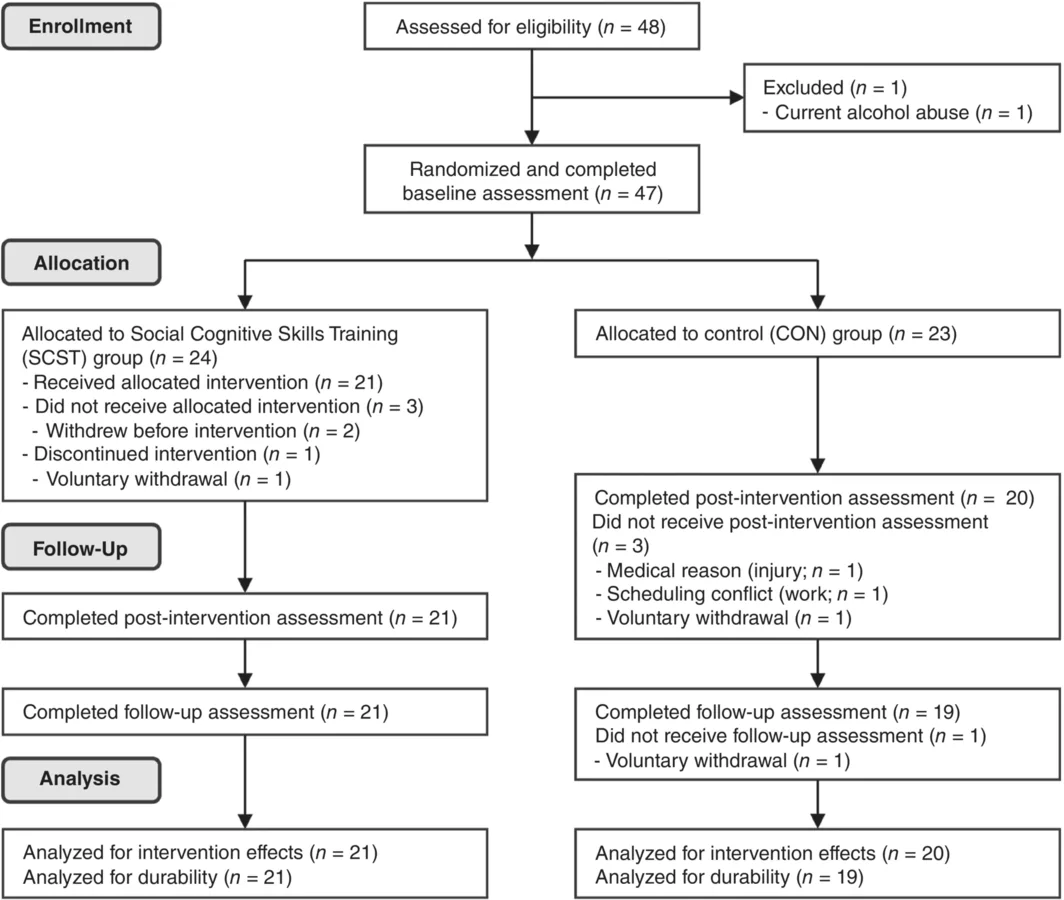
Flowchart of participant progress through the study.

The SCST intervention comprised twelve 1‐h sessions conducted over 6 weeks. Sessions could be postponed by 1 week for unavoidable reasons (e.g., national holidays, facility closures). Two sessions were held on the same day, separated by a short break, at the outpatient facilities participants regularly attended. Groups comprised four to six participants and were led by two to three co‐facilitators. The facilitators were one of two occupational therapists (second and third authors), supervised by a clinical psychologist with expertise in cognitive training interventions (first author) and accompanied by facility staff. Staff members were licensed clinical psychologists, occupational therapists, mental health social workers, or psychiatric nurses. All participants continued to receive treatment as usual, comprising standard outpatient psychiatric care, including pharmacotherapy and routine clinical management. Because participants were recruited from outpatient psychiatric rehabilitation settings, this care also involved rehabilitation programs, such as group activities, occupational therapy, and social rehabilitation. However, it did not include structured interventions specifically targeting social cognition. The SCST group participated in SCST as part of their regular rehabilitation program; the CON group attended other facility‐based programs. There were no significant between‐group differences in total facility use hours during the intervention (*P* = 0.231; Table 1).

The study protocol was approved by the Ethics Committee of the Graduate School and Faculty of Medicine, Kyoto University (approval code: C1473; May 19, 2020), and preregistered on the University Hospital Medical Information Network Clinical Trials Registry (UMIN000041619; https://center6.umin.ac.jp/cgi-open-bin/ctr/ctr_view.cgi?recptno=R000047105; September 7, 2020). All procedures adhered to the Declaration of Helsinki (1964) and subsequent amendments. Written informed consent was obtained from all participants.

### Intervention

We developed and implemented a Japanese version of the 6‐week SCST.^26^ As the structure and content of SCST have been previously described,^23^, ^24^, ^25^ only a brief summary is provided here.

The program progresses from lower‐level to higher‐level social cognition functions. The first eight sessions focus on emotion perception: the first four address facial expressions and the subsequent four address nonverbal gestures and vocal cues. The final four sessions target theory of mind, emphasizing empathic understanding of others' mental states. Each session included a review of previous content, introduction of new cognitive strategies, and structured practice exercises. Training incorporated strategy‐building techniques frequently used in psychiatric rehabilitation, such as breaking down complex social cognitive processes into component strategies and reinforcing them through repetition and feedback. All sessions were delivered using PowerPoint presentations. The facilitator manual and all training materials were translated and culturally adapted into Japanese by two clinical psychologists (including the first author), with permission from the original developers. Using translated scripts, 21 video clips were produced featuring Japanese actors demonstrating empathic and non‐empathic listening. The actors and camera operator were members of a university theater group.

Homework assignments were newly developed for the Japanese SCST. These were designed to promote skill generalization to daily life and were informed by strategy‐based approaches to neurocognitive remediation for SCZ.^32^, ^33^ The assignments encouraged participants to practice making and interpreting facial expressions using mirrors or media examples, identify emotional cues in television/film characters, and apply empathic listening strategies in real‐life interactions. At the end of each session, participants and facilitators jointly planned how to implement the new strategies in everyday contexts (i.e., when, where, to whom, and how). Homework planning sheets included tips for prospective memory support (e.g., linking assignments to routines or using reminders). During the final session, participants developed individualized plans for continued practice after the program to maintain and generalize training effects.

To monitor treatment fidelity, two or more facilitators rated each session on 10 implementation items using a 3‐point scale (0 = not done, 1 = partially done, 2 = fully done): (1) sharing and generalizing previous homework, (2) troubleshooting homework difficulties, (3) introducing new strategies, (4) explaining the rationale for strategies, (5) demonstrating exercises, (6) providing positive feedback, (7) providing constructive feedback, (8) assigning homework, (9) supporting homework completion, and (10) managing time appropriately. Mean adherence ratings exceeded 1.64 across all items, indicating strong protocol fidelity.

### Impact of COVID‐19

All interventions were conducted between November 2020 and March 2022. The program was not interrupted by the COVID‐19 pandemic; however, participants and facilitators had to wear masks in accordance with infection control protocols. Nonwoven masks were initially used, but were later replaced by clear plastic masks to ensure that facial expressions were visible, which was essential to the training.

### Measures

Table 2 summarizes the outcome measures. The primary outcomes focused on social cognition and included two measures of facial emotion perception (the Facial Emotion Recognition: FER; and the Facial Emotion Recognition through Brief Presentation: FER‐BP), one of multisensory emotion perception (the Reading the Mind in Films test: RMF), one of emotion management (the Managing Emotions subtest of the Mayer‐Salovey‐Caruso Emotional Intelligence Test: MSCEIT‐ME), and one of theory of mind (the Reading the Mind in the Eyes test: Eyes test). FER, FER‐BP, RMF, and MSCEIT‐ME assessed lower‐level social cognitive domains, whereas the Eyes test assessed a higher‐level domain. None of the stimuli used in these tasks appeared in the training sessions. The secondary outcomes assessed neurocognition, clinical symptoms, and daily and social functioning. Neurocognition was assessed using the MATRICS Consensus Cognitive Battery (MCCB), and clinical symptoms were assessed using the Positive and Negative Syndrome Scale (PANSS) for SCZ and the Childhood Autism Rating Scale, Second edition, High Functioning version (CARS2‐HF) for ASD. Functional outcomes included social functioning, assessed using the Social Outcome Composite Rating (SOCR), quality of life, assessed using the World Health Organization Quality of Life‐26 (WHOQOL‐26), and global functioning, assessed using the Global Assessment of Functioning (GAF). Additional exploratory self‐report outcomes were described in Table S2. In addition, an exploratory eye‐tracking measure was introduced during the trial; however, the eye‐tracking data are not reported here because the sample size was insufficient for the present analyses. Harms were not anticipated and were assessed non‐systematically. No adverse events occurred.

**Table 2:** Descriptions of outcome measures.

Domain	Task	Description	Measure
Primary outcomes: social cognition			
Emotion perception (facial emotion perception)	Facial Emotion Recognition task (FER)	The FER task, which we used in a previous study,^12^ assesses the ability to recognize six basic emotions (anger, disgust, fear, happiness, sadness, and surprise) from facial expressions. It comprises 24 photographs of young adult faces from a standardized set^34^ and presented on a computer monitor for 2000 ms. After each photograph disappears, six emotion labels appear and participants must select the one that best matches the expression within 10 s. Each expression was presented four times under two intensity conditions (60% and 100%), with equal numbers of Japanese and Caucasian faces. Two alternative versions of the task were administered in a counterbalanced order across participants, and the same set of photographs was used at baseline and follow‐up.	Number of correct responses (0–24)
(Automatic processing of facial expressions)	Facial Emotion Recognition through Brief Presentation (FER‐BP)	Before the FER task, participants completed the FER‐BP task, also used in our previous study,^12^ which assesses recognition of six basic emotions from briefly presented facial expressions. This task is identical to the FER except for the shorter presentation time (50 ms), which is intended to minimize the use of compensatory strategies and ensure reliance on automatic processing. The same photographs were used in a different order, and two alternative versions were counterbalanced across participants. After the FER tasks, participants performed a face perception task to confirm whether they could perceive faces presented for 50 ms. In this task, participants had to view 12 natural‐expression faces for 50 ms and then choose which of two neutral faces had just been shown, within 10 s. Because the vast majority of participants (83.6%) answered all items correctly and only one participant made more than 3 errors (5 at baseline, 7 at post‐intervention, and 4 at follow‐up), the data from that participant were excluded from the analyses.	Number of correct responses (0–24)
(Multisensory emotion perception)	Reading the Mind in Films test (RMF)	The RMF^35^ assesses the ability to infer others' emotional states from short movie scenes that provide both visual and auditory social cues. It consists of 22 brief dialogue scenes, each presented with four adjectives, from which participants select the one that best describes the specified character's feeling at the end of the scene. One point is awarded for each correct response.	Number of correct responses (0–22)
Theory of mind	Reading the Mind in the Eyes test (Eyes test)	The Eyes test^36^ assesses the ability to infer others' mental states from subtle cues in the eye region. It consists of 36 photographs of eyes, each accompanied by four adjectives, from which participants select the one that best describes the depicted mental state. One point is given for each correct response.	Number of correct responses (0–36)
Emotion management	Managing Emotions subtest of the Mayer‐Salovey‐Caruso Emotional Intelligence Test (MSCEIT‐ME)	The MSCEIT‐ME^37^ in the MATRICS Consensus Cognitive Battery (MCCB)^38^ assesses the ability to regulate emotions in oneself and in interactions with others. It consists of 29 scenario‐based items involving decision‐making in emotional situations. Participants rate the effectiveness of each decision on a 5‐point scale. Total scores were calculated using the general consensus method based on a large normative sample.	Standardized score (Mean = 100, Standard deviation = 15)
Secondary outcomes			
Neurocognition	MATRICS Consensus Cognitive battery (MCCB)	Neurocognition was assessed using the six neurocognitive domains of the MCCB^38^: speed of processing (BACS Symbol Coding, Category Fluency, Trail Making Test‐A), attention/vigilance (CPT‐IP), working memory (WMS‐III Spatial Span, Letter‐Number Span), verbal learning (HVLT‐R), visual learning (BVMT‐R), and reasoning and problem solving (NAB Mazes).	Sum of T‐scores across six domains
Clinical symptoms (Schizophrenia: SCZ)	Positive and Negative Syndrome Scale (PANSS)	The severity of clinical symptoms in participants with SCZ was measured using the PANSS.^39^ This scale consists of 30 items that assess symptom severity across three domains: positive symptoms, negative symptoms, and general psychopathology. Each item is rated on a scale from 1 to 7; higher scores indicate greater symptom severity.	Total raw score (30–210)
(Autism spectrum disorder: ASD)	Childhood Autism Rating Scale, Second edition, High Functioning version (CARS2‐HF)	The severity of clinical symptoms in participants with ASD was measured using the CARS2‐HF.^40^ This consists of 15 items that assess autism‐related behaviors. Each item is rated on a scale from 1.0 to 4.0, with higher scores indicating greater symptom severity.	Total raw score (15–60)
Schizotypal traits	Schizotypal Personality Questionnaire (SPQ)	The Japanese version of the SPQ^41^, ^42^ assesses schizotypal traits. This self‐report questionnaire consists of 74 items requiring “yes” or “no” responses. Responses reflecting schizotypal traits were scored as one point, with higher scores indicating stronger schizotypal traits.	Total raw score (0–74)
Autistic traits	Autism Spectrum Quotient (AQ)	The AQ^43^, ^44^ assesses autistic traits. This self‐report questionnaire consists of 50 items rated on a 4‐point scale. Responses reflecting autistic traits were scored as one point, with higher scores indicating stronger autistic traits. It has also been reported that, in schizophrenia, AQ scores may reflect autism‐like social dysfunctions rather than innate autistic traits.^22^	Total raw score (0–50)
Social functioning	Social Outcome Composite Rating (SOCR)	Social functioning was assessed using a composite rating derived from four domains: residential status, employment/education, intimate relationships, and friendships. Following previously established criteria for adults with ASD,^12^ which were based on pioneering studies,^45^, ^46^ one point was assigned for each domain fulfilled: living independently or with a spouse/children; working >10 h per week or attending postsecondary education; being married or in a relationship lasting >1 year; and having met at least one friend within the last 3 months.	Number of domains achieved (0–4)
Quality of life	World Health Organization Quality of Life‐26 (WHOQOL‐26)	The Japanese version of the WHOQOL‐26 (also known as WHOQOL‐BREF)^47^, ^48^ assesses subjective quality of life. This self‐report questionnaire consists of 26 items rated on a 5‐point scale, with higher scores indicating higher quality of life.	Mean score across all items (1–5)
Global functioning	Global Assessment of Functioning (GAF)	The GAF^49^, ^50^ assesses psychological, social, and occupational functioning. Based on participant interviews, functioning was rated on a scale from 0 to 100, with higher scores indicating better global functioning.	Raw score (0–100)

### Feasibility ratings

At the end of each session, participants rated on a 0–100 visual analog scale how much they (1) enjoyed the session, (2) actively participated, and (3) found the training helpful to their daily life.

### Statistical analyses

All analyses were conducted using R version 4.5.0 (R Foundation for Statistical Computing, Vienna, Austria). Two‐tailed tests were used with α = 0.05.

Preliminary analyses: baseline differences in demographic, clinical, and intellectual variables between the SCST and CON groups were examined to determine potential covariates.

Primary efficacy analyses: intervention effects were evaluated using generalized linear mixed models (GLMMs) to examine group differences in longitudinal trajectories across time and diagnosis (SCZ vs ASD). Analyses followed the intention‐to‐treat principle, using all available data under the assumption of missing completely at random.^51^, ^52^ Fixed effects included group (SCST, CON), diagnosis (ASD, not ASD), time (0w, 6w), and their interactions (group × time, group × time × diagnosis). Subject‐level random intercepts were used to account for repeated measures. The significance of fixed effects was tested using *F*‐tests with Kenward–Roger degrees‐of‐freedom correction, which provides more conservative degrees of freedom and can reduce the probability of type I errors.^53^ Significant interactions were examined using pairwise comparisons within each group, or within each diagnostic category for three‐way interactions.

Generalization analyses: changes in social and global functioning from baseline (0w) to follow‐up (12w) were examined using GLMMs to evaluate whether functional gains were observed post‐intervention. In addition, simple regression was used to test whether improvements in specific social cognitive domains (0–6w) predicted subsequent changes in functioning (0–12w) across all participants and within each diagnostic group.

Durability analyses: For outcomes showing significant group × time or group × time × diagnosis effects during intervention, we assessed trajectories from 6 to 12 weeks using GLMMs. Lack of significant interactions indicated maintenance of effects. In addition, trajectories from 0 to 12 weeks were analyzed to confirm maintenance of improvements over the entire study period.

The following supplementary analyses were conducted to explore possible explanations for the disorder‐specific effects of SCST. First, we compared baseline demographic, clinical, intellectual, cognitive, and functional characteristics between participants with SCZ and those with ASD. Next, for outcome measures that showed significant interactions, we reanalyzed the effects, including as covariates any demographic or intellectual variables that differed significantly between the two diagnostic groups. Finally, to explore potential differences in the mechanisms underlying social cognitive impairments, we calculated Pearson correlations between baseline social cognition and neurocognition scores separately for each disorder.

## Results

### Feasibility of intervention

Attendance for the SCST sessions was excellent; the mean attendance rate was 92.2%. Retention was high: Attrition at post‐intervention was 12.5% (3/24) in the SCST group (two withdrew before starting and one discontinued) and 13.0% (3/23) in the CON group; by follow‐up, attrition remained 12.5% in SCST and increased to 17.4% in CON (see Fig. 1). Participants reported positive experiences with the program; the mean ratings on the 0–100 visual analog scale were 75.0 for enjoyment, 74.2 for active participation, and 73.6 for perceived usefulness for daily life.

### Effects of the intervention

No significant baseline differences were observed between the SCST and CON groups in demographic, clinical, or intellectual characteristics, including hours spent in rehabilitation programs (all *P* ≥ 0.125; Table 1). Therefore, the analyses proceeded without covariate adjustment. The GLMM results are summarized in Table 3, and descriptive statistics with effect sizes by diagnosis are provided in Table S3.

**Table 3:** Intervention effects: Estimated marginal means across assessments.

	SCST, mean (SE)	CON, Mean (SE)	Group	Time	G. × T. interaction	G. × T. × D. interaction
Social cognition: primary outcome						
FER						
Baseline	15.94 (0.60)	14.97 (0.60)	*F*(1,60.40) = 0.66 *P* = 0.420 *f* ^2^ = 0.01	*F*(1,40.98) = 15.07 *P* < 0.001 *f* ^2^ = 0.37	*F*(1,40.98) = 5.64 *P* = 0.022 *f* ^2^ = 0.14	*F*(1,40.98) = 2.38 *P* = 0.131 *f* ^2^ = 0.06
Post‐intervention	18.88 (0.64)	15.68 (0.64)				
FER‐BP						
Baseline	13.74 (0.68)	12.56 (0.68)	*F*(1,62.93) = 0.51 *P* = 0.478 *f* ^2^ < 0.01	*F*(1,36.96) = 7.86 *P* = 0.008 *f* ^2^ = 0.21	*F*(1,36.96) < 0.01 *P* = 0.977 *f* ^2^ = 0.03	*F*(1,36.96) = 2.55 *P* = 0.119 *f* ^2^ = 0.07
Post‐intervention	15.07 (0.72)	13.87 (0.71)				
RMF						
Baseline	12.70 (0.57)	12.54 (0.57)	*F*(1,79.93) = 1.96 *P* = 0.166 *f* ^2^ = 0.02	*F*(1,38.29) < 0.01 *P* = 0.960 *f* ^2^ < 0.01	*F*(1,38.29) = 7.07 *P* = 0.011 *f* ^2^ = 0.18	*F*(1,38.29) = 11.13 *P* = 0.002 *f* ^2^ = 0.29
Post‐intervention	12.06 (0.58)	13.20 (0.59)				
Eyes test						
Baseline	20.91 (0.75)	18.25 (0.76)	*F*(1,65.20) = 2.73 *P* = 0.103 *f* ^2^ = 0.04	*F*(1,39.72) < 0.01 *P* = 0.961 *f* ^2^ < 0.01	*F*(1,39.72) = 0.09 *P* = 0.771 *f* ^2^ < 0.01	*F*(1,39.72) = 5.02 *P* = 0.031 *f* ^2^ = 0.13
Post‐intervention	20.73 (0.80)	18.38 (0.80)				
MSCEIT‐ME in MCCB						
Baseline	84.64 (1.92)	80.84 (1.94)	*F*(1,74.82) = 0.36 *P* = 0.550 *f* ^2^ < 0.01	*F*(1,39.40) = 0.81 *P* = 0.374 *f* ^2^ = 0.02	*F*(1,39.40) = 0.39 *P* = 0.534 *f* ^2^ < 0.01	*F*(1,39.40) = 0.06 *P* = 0.804 *f* ^2^ < 0.01
Post‐intervention	86.32 (2.01)	81.14 (2.01)				
Neurocognition						
MCCB						
Baseline	267.12 (10.49)	264.14 (10.57)	*F*(1,71.85) < 0.01 *P* = 0.986 *f* ^2^ < 0.01	*F*(1,37.55) = 6.82 *P* = 0.013 *f* ^2^ = 0.18	*F*(1,37.55) = 0.25 *P* = 0.622 *f* ^2^ < 0.01	*F*(1,37.55) = 0.17 *P* = 0.685 *f* ^2^ < 0.01
Post‐intervention	277.39 (10.64)	271.13 (10.71)				
Clinical symptoms						
PANSS						
Baseline	66.08 (3.42)	59.50 (3.42)	*F*(1,29.59) = 2.36 *P* = 0.135 *f* ^2^ = 0.08	*F*(1,20.00) = 0.17 *P* = 0.683 *f* ^2^ < 0.01	*F*(1,20.00) = 1.02 *P* = 0.324 *f* ^2^ = 0.05	
Post‐intervention	65.09 (3.44)	59.92 (3.42)				
CARS2‐HF						
Baseline	35.86 (0.99)	33.42 (1.07)	*F*(1,37.70) = 3.53 *P* = 0.068 *f* ^2^ = 0.09	*F*(1,20.01) = 8.40 *P* = 0.009 *f* ^2^ = 0.42	*F*(1,20.01) = 1.32 *P* = 0.264 *f* ^2^ = 0.07	
Post‐intervention	34.66 (0.99)	32.90 (1.09)				
SPQ						
Baseline	40.31 (3.29)	35.26 (3.31)	*F*(1,71.22) = 1.25 *P* = 0.267 *f* ^2^ = 0.02	*F*(1,37.65) = 2.04 *P* = 0.162 *f* ^2^ = 0.05	*F*(1,37.65) = 1.77 *P* = 0.257 *f* ^2^ < 0.01	*F*(1,37.65) = 1.31 *P* = 0.261 *f* ^2^ = 0.03
Post‐intervention	38.34 (3.33)	34.32 (3.36)				
AQ						
Baseline	29.74 (1.48)	28.73 (1.49)	*F*(1,79.93) = 0.01 *P* = 0.930 *f* ^2^ < 0.01	*F*(1,38.31) = 0.07 *P* = 0.794 *f* ^2^ < 0.01	*F*(1,38.31) = 0.31 *P* = 0.579 *f* ^2^ < 0.01	*F*(1,38.31) = 0.28 *P* = 0.600 *f* ^2^ < 0.01
Post‐intervention	30.29 (1.52)	28.53 (1.53)				
Functioning						
SOCR						
Baseline	1.19 (0.19)	1.08 (0.20)	*F*(1,78.95) = 0.68 *P* = 0.410 *f* ^2^ < 0.01	*F*(1,36.80) = 0.14 *P* = 0.715 *f* ^2^ < 0.01	*F*(1,36.80) = 5.70 *P* = 0.022 *f* ^2^ = 0.15	*F*(1,36.80) = 1.62 *P* = 0.211 *f* ^2^ = 0.04
Follow‐up	1.36 (0.20)	0.85 (0.20)				
WHOQOL‐26						
Baseline	2.74 (0.12)	2.79 (0.12)	*F*(1,78.57) = 0.82 *P* = 0.368 *f* ^2^ = 0.01	*F*(1,37.49) = 3.98 *P* = 0.053 *f* ^2^ = 0.11	*F*(1,37.49) = 1.77 *P* = 0.192 *f* ^2^ = 0.05	*F*(1,37.49) = 0.74 *P* = 0.397 *f* ^2^ = 0.02
Follow‐up	2.92 (0.12)	2.83 (0.12)				
GAF						
Baseline	48.71 (1.65)	48.32 (1.66)	*F*(1,70.86) = 0.13 *P* = 0.721 *f* ^2^ < 0.01	*F*(1,36.54) = 0.29 *P* = 0.595 *f* ^2^ < 0.01	*F*(1,36.54) = 1.76 *P* = 0.193 *f* ^2^ = 0.05	*F*(1,36.54) = 5.29 *P* = 0.027 *f* ^2^ = 0.14
Follow‐up	49.67 (1.67)	47.91 (1.69)				

*n* = 47 (FER‐BP: *n* = 46; PANSS: *n* = 24; CARS2‐HF: *n* = 26).

AQ, autism spectrum quotient; CARS2‐HF, childhood autism rating scale, second edition, high‐functioning version; CON, control; Eyes test, reading the mind in the eyes test; FER, facial emotion recognition task; FER‐BP, facial emotion recognition through brief presentation task; G. × T. × D., group by time by diagnosis; G. × T., group by time; GAF, global assessment of functioning; MCCB, MATRICS consensus cognitive battery; MSCEIT‐ME, managing emotions subtest of the Mayer‐Salovey‐Caruso emotional intelligence test; PANSS, positive and negative syndrome scale; RMF, reading the mind in films test; SCST, social cognitive skills training; SE, standard error; SOCR, social outcome composite rating; SPQ, schizotypal personality questionnaire; WHOQOL‐26, World Health Organization quality of life‐26.

#### Primary outcomes: social cognition

A significant group × time interaction emerged for facial emotion recognition, as measured by the FER (*P* = 0.022), but not for automatic emotion processing, as measured by the FER‐BP (*P* = 0.977). *Post hoc* tests showed a significant improvement in FER performance from baseline (0w) to post‐intervention (6w) only in the SCST group (*t*(38.17) = 1.82, *P* = 0.008; Fig. 2a). For multisensory emotion perception, a significant group × time × diagnosis interaction was observed on the RMF (*P* = 0.002). Among participants with SCZ, RMF performance declined during the intervention in the SCST group (*t*(38.17) = −2.90, *P* = 0.006) but improved in the CON group (*t*(37.03) = 2.91, *P* = 0.006; Fig. 2b). No significant SCST effects were found for emotion management on the MSCEIT‐ME (*P* = 0.534).

**Fig. 2:**
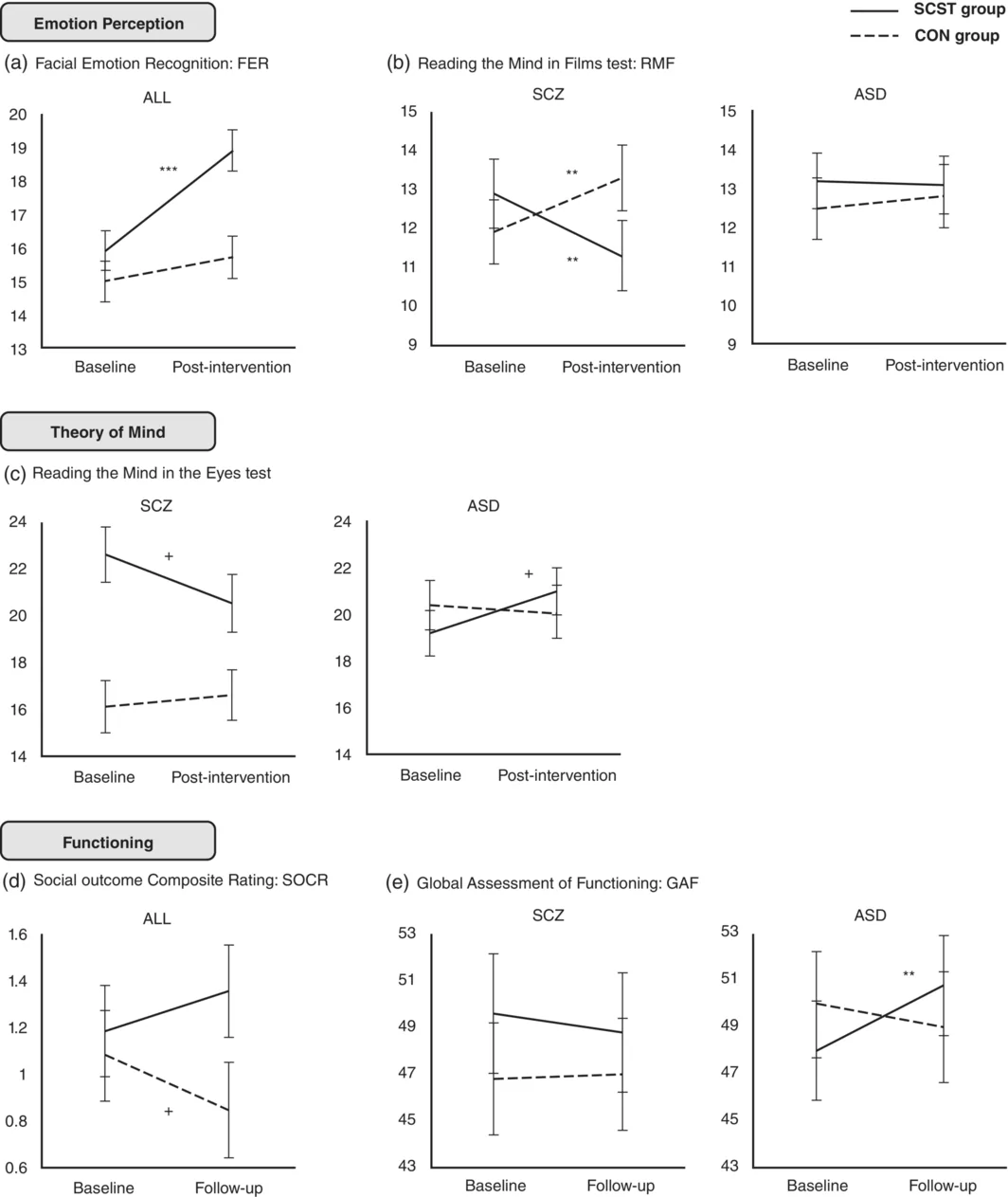
Intervention effects: Estimated marginal means across assessments for measures showing significant effects. Changes in social cognition (from baseline to post‐intervention) and functioning (from baseline to follow‐up) across the intervention (SCST) and control (CON) groups. (a) Significant group × time interaction indicating improved facial emotion perception following SCST across diagnoses. (b) Significant group × time × diagnosis interaction; among participants with schizophrenia (SCZ), multisensory emotion perception declined in the SCST group but improved in the CON group. (c) Significant group × time × diagnosis interaction; participants with autism spectrum disorder (ASD) in SCST showed trend‐level improvement in theory of mind, whereas SCZ participants in SCST showed a decline. (d) Significant group × time interaction indicating maintained social functioning in SCST and a trend‐level decline in CON. (e) Significant group × time × diagnosis interaction; ASD participants in SCST showed significant improvement at follow‐up. Error bars represent standard errors. Solid lines = SCST group; dashed lines = CON group. ^+^ *P* < 0.10, *P < 0.05, **P < 0.01, ***P < 0.001.

For theory of mind (higher‐level), a significant group × time × diagnosis interaction was found on the Eyes test (*P* = 0.031). *Post hoc* analyses indicated a trend‐level decline in SCZ participants receiving SCST (*t*(40.04) = −1.82, *P* = 0.077) and a trend‐level improvement in ASD participants receiving SCST (*t*(38.19) = 1.90, *P* = 0.065; Fig. 2c).

### Secondary outcomes

No significant intervention effects were found for neurocognition (*P* = 0.622) or clinical symptoms (all *P* ≥ 0.257). However, for social functioning, a significant group × time interaction emerged for the SOCR (*P* = 0.022). *Post hoc* comparisons identified a trend‐level decline from baseline (0w) to follow‐up (12w) in the CON group only (*t*(37.08) = −1.92, *P* = 0.063; Fig. 2d). Additionally, a significant group × time × diagnosis interaction was found on the GAF (*P* = 0.027). *Post hoc* tests indicated significant improvement from baseline to follow‐up in the SCST group for ASD participants only (*t*(36.21) = 3.09, *P* = 0.004; Fig. 2e). No significant intervention effects were observed for the additional exploratory self‐report outcomes (Table S4).

We conducted supplementary simple regression analyses to test whether gains in social cognition predicted functional improvement. Improvement in FER from 0 to 6 weeks predicted improvement in GAF from 0 to 12 weeks across all participants (*t*(38) = 2.06, *P* = 0.047, *β* = 0.32) and for ASD participants (*t*(19) = 2.87, *P* = 0.010, *β* = 0.56), but not SCZ participants (*t*(17) = −1.05, *P* = 0.310, *β* = −0.25). Similarly, FER improvement predicted SOCR gains in ASD (*t*(19) = 2.52, *P* = 0.021, *β* = 0.51) but not SCZ (*t*(17) = −0.63, *P* = 0.535, *β* = 0.15).

### Durability of effects

Durability analyses focused on the three social cognitive measures showing significant interactions (FER, RMF, and Eyes test; see Table 4). From post‐intervention (6w) to follow‐up (12w), no significant changes were found on FER or the Eyes test (all *P* ≥ 0.561; Fig. 3a,c), suggesting that gains were maintained. Comparing baseline (0w) to follow‐up (12w), significant effects were again observed, with a group × time interaction for FER (*P* < 0.001) and a group × time × diagnosis interaction for the Eyes test (*P* = 0.014), replicating earlier patterns. *Post hoc* comparisons identified a trend‐level decline in SCST participants with SCZ (*t*(38.36) = −1.80, *P* = 0.080) and a trend‐level improvement in SCST participants with ASD (*t*(36.92) = 2.02, *P* = 0.050). Together, these results demonstrate the durability of the observed improvements in facial emotion perception and theory of mind.

**Table 4:** Durability of effects: Estimated marginal means across assessments

	SCST, mean (SE)	CON, mean (SE)	Group	Time	G. × T. interaction	G. × T. × D. interaction
Post‐intervention to Follow‐up (*n* = 41)						
FER						
Post‐intervention	18.74 (0.71)	15.68 (0.71)	*F*(1,59.02) = 2.28 *P* = 0.136 *f* ^2^ = 0.04	*F*(1,36.77) = 0.26 *P* = 0.613 *f* ^2^ < 0.01	*F*(1,36.77) = 0.21 *P* = 0.647 *f* ^2^ < 0.01	*F*(1,36.77) = 0.34 *P* = 0.561 *f* ^2^ < 0.01
Follow‐up	18.72 (0.71)	15.19 (0.73)				
RMF						
Post‐intervention	11.82 (0.63)	13.41 (0.63)	*F*(1,72.24) = 6.87 *P* = 0.011 *f* ^2^ = 0.10	*F*(1,36.09) = 0.25 *P* = 0.618 *f* ^2^ < 0.01	*F*(1,36.09) = 7.25 *P* = 0.011 *f* ^2^ = 0.20	*F*(1,36.09) = 9.67 *P* = 0.004 *f* ^2^ = 0.27
Follow‐up	12.81 (0.63)	12.74 (0.64)				
Eyes test						
Post‐intervention	20.67 (0.79)	18.26 (0.79)	*F*(1,67.54) = 2.87 *P* = 0.095 *f* ^2^ = 0.04	*F*(1,36.61) = 0.52 *P* = 0.517 *f* ^2^ = 0.01	*F*(1,36.61) = 0.09 *P* = 0.771 *f* ^2^ < 0.01	*F*(1,36.61) = 0.12 *P* = 0.735 *f* ^2^ < 0.01
Follow‐up	20.74 (0.79)	18.80 (0.80)				
Baseline to follow‐up (*n* = 47)						
FER						
Baseline	15.94 (0.62)	14.97 (0.62)	*F*(1,69.91) = 2.11 *P* = 0.151 *f* ^2^ = 0.03	*F*(1,38.72) = 15.13 *P* < 0.001 *f* ^2^ = 0.39	*F*(1,38.72) = 14.76 *P* < 0.001 *f* ^2^ = 0.38	*F*(1,38.72) = 0.75 *P* = 0.390 *f* ^2^ = 0.02
Follow‐up	18.92 (0.65)	14.99 (0.66)				
RMF						
Baseline	12.70 (0.57)	12.54 (0.57)	*F*(1,76.41) = 0.01 *P* = 0.924 *f* ^2^ < 0.01	*F*(1,38.05) = 0.40 *P* = 0.533 *f* ^2^ = 0.01	*F*(1,38.05) = 0.21 *P* = 0.651 *f* ^2^ < 0.01	*F*(1,38.05) = 0.29 *P* = 0.593 *f* ^2^ < 0.01
Follow‐up	13.02 (0.59)	12.59 (0.60)				
Eyes test						
Baseline	20.91 (0.81)	18.25 (0.81)	*F*(1,69.78) = 3.77 *P* = 0.056 *f* ^2^ = 0.05	*F*(1,38.92) = 0.37 *P* = 0.549 *f* ^2^ < 0.01	*F*(1,38.92) = 0.67 *P* = 0.418 *f* ^2^ = 0.02	*F*(1,38.92) = 6.68 *P* = 0.014 *f* ^2^ = 0.17
Follow‐up	20.80 (0.85)	18.97 (0.87)				

CON, control; Eyes test, reading the mind in the eyes test; FER, facial emotion recognition task; G. × T. × D., group by time by diagnosis; G. × T., group by time; RMF, reading the mind in films test; SCST, social cognitive skills training; SE, standard error.

**Fig. 3:**
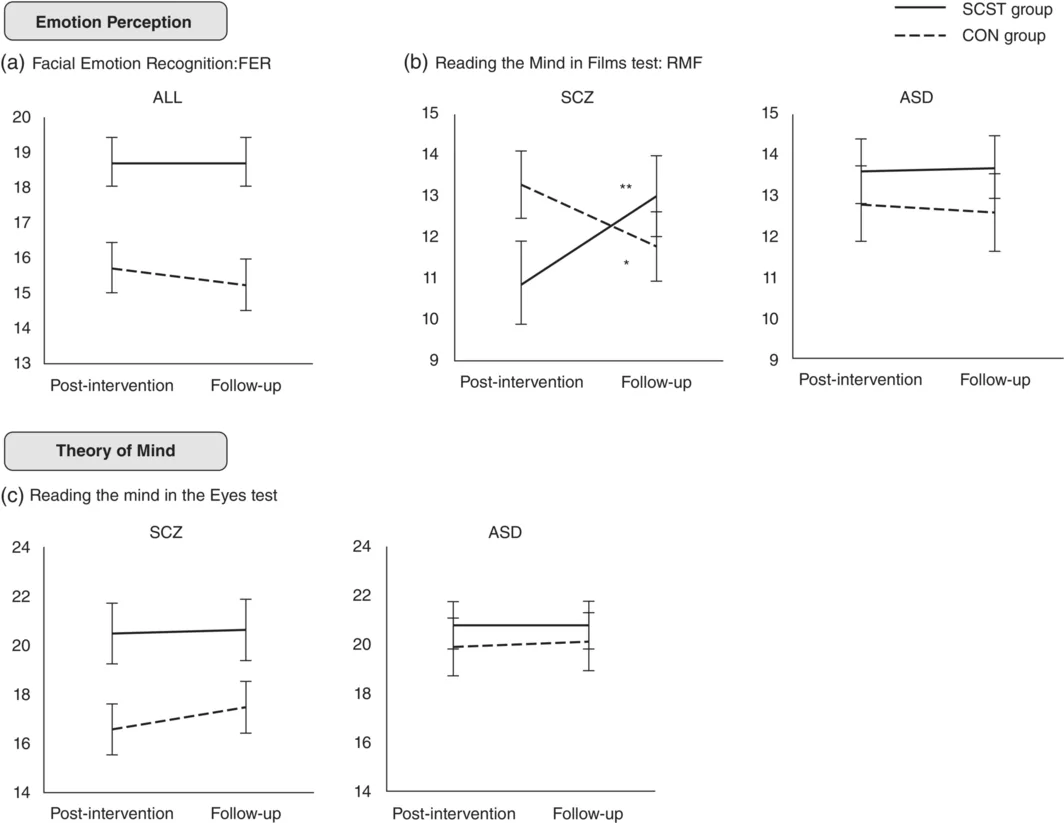
Durability of effects: Estimated marginal means across post‐intervention and follow‐up assessments for social cognition measures showing significant intervention effects. Changes in social cognition from post‐intervention to follow‐up across the social cognitive skills training (SCST) and control (CON) groups. (a) Performance gains in emotion perception produced by SCST were maintained at follow‐up. (b) Significant group × time × diagnosis interaction; among participants with schizophrenia (SCZ), multisensory emotion perception improved in SCST but declined in CON, returning to baseline by follow‐up. (c) No significant change between post‐intervention and follow‐up for either diagnosis, suggesting maintenance of theory of mind performance. Error bars represent standard errors. Solid lines = SCST group; dashed lines = CON group. *P < 0.05, **P < 0.01.

For RMF, a significant group × time × diagnosis interaction emerged between 6 weeks and 12 weeks (*P* = 0.004). *Post hoc* analyses showed improvement in SCST (*t*(36.01) = 3.14, *P* = 0.003) and decline in CON (*t*(36.01) = −2.52, *P* = 0.016) among participants with SCZ (Fig. 3b). When examined across the entire study period (0–12w), no significant changes were observed (all *P* ≥ 0.593).

### Supplementary analyses

At baseline, SCZ participants were significantly older and reported higher subjective quality of life than ASD participants, while ASD participants had higher Autism Spectrum Quotient (AQ) scores as expected (all *P* ≤ 0.024; see Table S5). No differences were found in intellectual or cognitive measures (all *P* ≥ 0.114). Reanalysis controlling for age confirmed that all significant group × time × diagnosis interactions remained robust (all *P* ≤ 0.031).

Finally, Pearson correlations between baseline social cognition and neurocognition were calculated separately for each disorder (see Tables S6 and S7). In SCZ, FER correlated strongly with estimated IQ (*r* = 0.65, *P* = 0.001) and MCCB composite (*r* = 0.68, *P* < 0.001), as well as multiple subdomains (all *P* ≤ 0.009). In ASD, FER correlated only with verbal working memory (*r* = 0.66, *P* = 0.001). Conversely, Eyes test performance correlated with estimated IQ (*r* = 0.51, *P* = 0.014) and MCCB composite (*r* = 0.53, *P* = 0.010) in ASD, but only with visual memory (*r* = 0.50, *P* = 0.021) and composite (*r* = 0.44, *P* = 0.047) in SCZ.

## Discussion

This RCT examined the feasibility and efficacy of the Japanese version of the 6‐week SCST in adults with SCZ and/or ASD. High attendance, low attrition across assessment time points, and favorable participant evaluations indicated strong feasibility and acceptability of this SCST as a transdiagnostic group intervention. We found that (i) SCST improved social cognition in both SCZ and ASD, particularly facial emotion perception, whereas no significant effects were observed for neurocognition or clinical symptoms, and (ii) this effect was maintained for at least 6 weeks after the intervention ended. (iii) Although these transdiagnostic improvements did not produce significant functional gains, (iv) additional ASD‐specific improvements were observed in theory of mind (trend‐level) and global functioning. To the best of our knowledge, this is the first RCT to demonstrate the feasibility and efficacy of broad‐based social cognition training for adults with ASD, as well as Japanese people with SCZ.

### Efficacy for schizophrenia

Extensive research has established the efficacy of SCST in improving social cognition in SCZ. We consider here how our findings align with and add to this evidence. The observed improvement in facial emotion perception replicates results from the original US trials^23^, ^24^, ^25^, ^26^ and from cross‐cultural adaptations.^54^, ^55^ Our study further contributes to this research by showing that a 6‐week SCST produced effects that were maintained at the 6‐week follow‐up. Notably, SCST improved performance on the FER but not the brief‐presentation version (FER‐BP), suggesting that it enhanced conscious, strategy‐based emotion recognition rather than automatic processing.

Performance on multisensory emotion perception (RMF) declined transiently during the intervention before recovering at follow‐up. This pattern likely reflects cognitive load from newly learned strategies, as the abbreviated SCST did not include integration sessions designed to unify multiple social cues. A similar short‐term decline was also seen for theory of mind (Eyes test). These findings may indicate the potential importance of integration sessions in social cognition training for SCZ, although this interpretation requires further confirmation.

Regarding secondary outcomes, SCST did not significantly improve social functioning. However, during the COVID‐19 pandemic, when social functioning in SCZ populations generally declined,^56^ social functioning in SCST participants was stable. These findings suggest that the homework assignments uniquely incorporated into the Japanese version may have encouraged real‐world application of learned strategies and supported their potential generalization. However, homework engagement was assessed qualitatively but not quantitatively (see the Intervention subsection in the Methods), and completion rates were not formally recorded, limiting conclusions about its specific contribution to maintenance or generalization. Consistent with previous findings, SCST did not improve neurocognition or clinical symptoms, emphasizing the specificity of its social cognitive focus. Overall, these results validate the Japanese adaptation of SCST as a culturally appropriate and effective intervention for social cognition in SCZ.

### Common effects in schizophrenia and autism

This study provides the first controlled evidence that a social cognition training program originally developed for SCZ can improve emotion perception in adults with ASD. Earlier pioneering studies^57^, ^58^, ^59^ suggested similar benefits but were limited by small (*n* = 10–11) and/or nonrandomized samples, which constrained their generalizability. Our RCT, which involved >20 adults with ASD, corroborates these early findings and extends them by demonstrating that a strategy‐based group training program can produce meaningful gains in emotion perception in this population, and that they can be maintained for at least 6 weeks post‐intervention.

Both diagnostic groups showed comparable baseline impairments in social cognition and neurocognition, consistent with meta‐analytic evidence indicating an overlap in cognitive deficits between SCZ and ASD.^8^ The parallel improvements in emotion perception across diagnoses suggest that SCST effectively targets shared cognitive mechanisms underlying social difficulties. Together, these findings support the transdiagnostic applicability of social cognition training and identify emotion perception as a fundamental, cross‐diagnostic treatment target in SCZ and ASD.

### Disorder‐specific effects on schizophrenia and autism

A key finding of this study was the ASD‐specific improvements in theory of mind (trend‐level) and global functioning, suggesting differential responsiveness to strategy‐based social cognitive interventions. Developmentally, theory of mind deficits in ASD emerge early^60^ and are linked to lifelong atypicalities in social inference,^61^ whereas in SCZ, they typically arise after disease onset.^21^ Individuals with ASD often compensate for, or camouflage,^62^ theory of mind deficits through language‐related cognitive strategies.^63^ Therefore, the explicit teaching of such strategies in this SCST intervention may have directly strengthened these compensatory mechanisms. In support of this interpretation, our supplementary correlation analyses showed that Eyes test performance in ASD, but not in SCZ, was positively associated with estimated IQ and performance on MCCB verbal tasks. In contrast, theory of mind in SCZ was more strongly linked to visual memory, suggesting reliance on different neural and cognitive systems. These differences likely account for the observed divergence in training effects, with higher‐order social cognition showing a tendency to improve among participants with ASD, possibly reflecting differences in developmental plasticity or habitual reliance on compensatory strategies.

Although SCST was originally developed for SCZ, it was associated with more favorable functional outcomes in participants with ASD, particularly for global functioning across social and psychological dimensions. This finding suggests that the cognitive gains from SCST may have generalized to broader functional benefits more effectively among individuals with ASD. Supplementary regression analyses further showed that improvements in emotion perception predicted gains in both social and global functioning for ASD participants but not for SCZ participants. This pattern indicates that emotion perception may be a more direct pathway to functional adaptation in ASD, whereas in SCZ, broader neurocognitive deficits or persistent negative symptoms may limit such generalization.

Because this study examined differences in treatment response between SCZ and ASD, it was important to carefully consider the possible influence of participants with comorbid SCZ and ASD. We therefore conducted a sensitivity analysis excluding these three cases. In this analysis, the group × time × diagnosis interaction for the Eyes test was no longer significant at post‐intervention (*P* = 0.054; Table S8), although it remained significant at follow‐up (*P* = 0.030). Although these three participants did not show markedly atypical baseline clinical or cognitive profiles (Table S9), this finding should be interpreted with caution. In contrast, for global functioning, the sensitivity analysis consistently suggested that the ASD‐specific improvement remained even after excluding the three comorbid cases (*P* = 0.024).

Taken together, these findings suggest that although transdiagnostic programs can effectively target shared social cognitive mechanisms, optimal outcomes may require diagnosis‐sensitive modules that address distinct developmental and cognitive pathways.

### Toward the development of hybrid and disorder‐sensitive interventions

The present findings suggest that an integrated framework combining transdiagnostic and disorder‐specific components may yield the greatest clinical benefit. The 6‐week SCST provides a solid foundation for transdiagnostic social cognition training, particularly in improving fundamental emotion perception. However, achieving optimal outcomes likely requires diagnosis‐sensitive modules that target higher‐order domains and foster the integrated application of social cognitive strategies. For SCZ, future adaptations could reintroduce integration sessions and include targeted modules focusing on theory of mind, such as structured group discussions of social vignettes that require participants to infer others' beliefs and emotions.^64^ For ASD, advanced training focused on resolving incongruent emotional cues (e.g., mismatched facial and vocal information) may enhance generalization by addressing atypical multisensory integration that interferes with accurate emotion interpretation.^65^, ^66^

Such hybrid models would allow clinicians to tailor intervention intensity and content to each diagnostic profile while retaining shared transdiagnostic components that support group‐based implementation and promote mutual social understanding among participants. Beyond their clinical utility, these frameworks may also deepen theoretical insights into both common and distinct neurocognitive mechanisms underlying social cognition across psychiatric conditions.

### Limitations

This study has several limitations. First, the relatively small sample size may limit the statistical power, particularly for detecting the three‐way interaction of group × time × diagnosis. This was reflected in the sensitivity analysis, in which the Eyes test finding was attenuated after excluding the three participants with comorbid SCZ and ASD. Although the GAF finding persisted in the sensitivity analysis, it was based on a secondary outcome in a relatively small exploratory trial and should therefore be interpreted cautiously. Because multiple outcomes were examined, the possibility of multiplicity should also be considered. Replication with larger samples is needed to confirm these findings. Second, the absence of an active CON group limited the ability to exclude nonspecific influences such as participant expectations, although the two groups did not differ in overall use of psychiatric rehabilitation services. Third, the follow‐up period was limited to 6 weeks post‐intervention; longer follow‐up is needed to evaluate the persistence of effects on social functioning. Fourth, the sample comprised adult outpatients with SCZ and/or ASD, which may restrict generalizability to other age groups or clinical settings, particularly to children with ASD. Finally, although the SCST program was carefully adapted for Japanese participants, cultural differences in social cognition may have affected the results; for example, Japanese adults reportedly rely more on vocal cues in emotion recognition than Western adults.^67^ Future research should develop and validate culturally sensitive social cognitive interventions that reflect such cross‐cultural differences.

## Conclusion

This study provides initial evidence supporting the feasibility and efficacy of social cognitive training as a transdiagnostic approach for adults with SCZ and/or ASD. The SCST intervention improved emotion perception in both populations, with durable effects lasting at least 6 weeks. In addition, the findings suggest possible benefits for theory of mind and global functioning in adults with ASD. These findings indicate that SCST offers a useful foundation for addressing shared impairments in basic social cognition across diagnostic boundaries. However, potential divergent responses in higher‐order domains suggest the need for diagnosis‐sensitive modules tailored to distinct developmental and cognitive characteristics. Future large‐scale studies should refine and expand these approaches by developing culturally sensitive, hybrid programs that combine core transdiagnostic elements with targeted components to enhance clinical outcomes and deepen understanding of the mechanisms underlying social cognitive impairments in psychiatric disorders.

## Author contributions

S.Ot conceived and designed the study; collected, analyzed, and interpreted the data; and drafted the manuscript. S.Oe, S.Y., H.K., R.S., T.I., and K.T. contributed to data acquisition. T.M. critically revised the manuscript for important intellectual content. All authors approved the final version of the manuscript and agreed to be accountable for all aspects of the work, ensuring that any questions related to the accuracy or integrity of any part of the study are appropriately investigated and resolved.

## Funding statement

This work was supported by JSPS KAKENHI (grant numbers JP18K13345 and JP23K22364). The funders had no role in the study design, data collection, data analysis, data interpretation, or writing of the manuscript.

## Disclosure statement

Author T.M. is a member of the Editorial Board of Psychiatry and Clinical Neurosciences. The remaining authors declare that they have no conflicts of interest.

## Ethics statement

The study protocol was approved by the Ethics Committee of the Graduate School and Faculty of Medicine, Kyoto University (approval code: C1473; May 19, 2020). All procedures adhered to the Declaration of Helsinki (1964) and subsequent amendments.

## Patient consent statement

Written informed consent was obtained from all participants.

## Supporting information


**Table S1.** Demographic and clinical data of SCZ and ASD participants.
**Table S2.** Descriptions of exploratory self‐report outcomes.
**Table S3.** Raw means of outcome measures for SCZ and ASD.
**Table S4.** Exploratory self‐reported outcomes: Estimated marginal means across assessments.
**Table S5.** Baseline comparison between SCZ and ASD.
**Table S6.** Baseline Pearson correlations between neurocognition and social cognition in SCZ.
**Table S7.** Baseline Pearson correlations between neurocognition and social cognition in ASD.
**Table S8.** Sensitivity analysis excluding participants with comorbid SCZ and ASD: Estimated marginal means across assessments.
**Table S9.** Baseline characteristics of participants with comorbid SCZ and ASD relative to participants with SCZ only and ASD only.

**Data S1.** CONSORT 2025 checklist of information to include when reporting a randomised trial*.

## Data Availability

The data that support the findings of this study are available on request from the corresponding author. The data are not publicly available due to privacy or ethical restrictions.
