# Supplementary material for: Different effects of social cognitive skills training in schizophrenia and autism spectrum disorder: A rater‐blinded, multicenter, randomized controlled trial
Source: Psychiatry Clin Neurosci. 2026 Jul 22;80(10):830–44. doi: 10.1111/pcn.70106 (PMC13644380; doi:10.1111/pcn.70106)
Supplement: Supplementary file 1 [file PCN-80-830-s001.docx]

**Supplementary materials**

**Table S1. Demographic and clinical data of SCZ and ASD participants**

|  | SCZ | |  | ASD | |  |
| --- | --- | --- | --- | --- | --- | --- |
|  | SCST (*n* = 10)  Mean (SD) | CON (*n* = 11)  Mean (SD) |  | SCST (*n* = 14)  Mean (SD) | CON (*n* = 12)  Mean (SD) |  |
| Age (years) | 49.90 (8.61) | 44.82 (9.77) | *t*(19) = 1.26, *p* = .223 | 38.43 (9.43) | 31.75 (11.44) | *t*(24) = 1.63, *p* = .116 |
| Education (years) | 14.70 (2.79) | 13.18 (2.04) | *t*(19) = 1.43, *p* = .168 | 14.36 (2.27) | 14.42 (2.15) | *t*(24) = 0.07, *p* = .946 |
| Duration (years) | 20.80 (11.20) | 21.09 (9.30) | *t*(19) = -0.07, *p* = .949 | 18.00 (*n* = 2) | 8.00 (*n* = 1) |  |
| Sex (Male%) | 20.00 | 45.45 | *χ*^2^(1) = 1.51, *p* = .217 | 78.57 | 66.67 | *χ*^2^(1) = 0.47, *p* = .495 |
| Dosage (mg/d, RPD eq.) | 4.99 (3.50) | 4.47 (2.94) | *t*(19) = 0.37, *p* = .713 | 1.71 (2.36) | 0.97 (1.26) | *t*(24) = 0.97, *p* = .344 |
| PANSS positive | 15.50 (3.41) | 13.55 (3.80) | *t*(19) = 1.24, *p* = .232 | 13.50 (*n* = 2) | 13.00 (*n* = 1) |  |
| PANSS negative | 18.40 (4.81) | 17.00 (4.22) | *t*(19) = 0.71, *p* = .486 | 15.00 (*n* = 2) | 17.00 (*n* = 1) |  |
| CARS2-HF | NA | NA |  | 35.86 (4.74) | 33.42 (2.57) | *t*(24) = 1.59, *p* = .125 |
| SPQ | 38.40 (13.88) | 33.18 (20.11) | *t*(19) = 0.68, *p* = .502 | 37.33 (9.82) | 42.21 (17.91) | *t*(24) = 0.84, *p* = .409 |
| AQ | 25.55 (7.85) | 27.90 (7.98) | *t*(19) = 0.68, *p* = .504 | 31.57 (5.03) | 31.92 (6.84) | *t*(24) = 0.15, *p* = .884 |
| JART-estimated-IQ | 111.10 (8.08) | 106.55 (10.73) | *t*(19) = 1.09, *p* = .289 | 108.07 (9.34) | 108.17 (5.02) | *t*(24) = -0.03, *p* = .975 |
| WAIS-III-estimated-IQ | 97.20 (20.09) | 92.00 (17.09) | *t*(19) = 0.64, *p* = .529 | 97.29 (26.46) | 96.17 (15.41) | *t*(24) = 0.13, *p* = .899 |
| Rehabilitation time (h/w) | 4.68 (4.74) | 3.50 (3.46) | *t*(19) = 0.66, *p* = .520 | 7.21 (4.50) | 5.52 (4.97) | *t*(24) = 0.91, *p* = .373 |

Note. SCZ = schizophrenia, ASD = autism spectrum disorder (the ASD category includes 3 participants with comorbid SCZ), SCST = social cognitive skills training, CON = control, SD = standard deviation, RPD eq. = risperidone equivalence, PANSS = Positive and Negative Syndrome Scale, CARS2-HF = Childhood Autism Rating Scale, Second edition, High Functioning version, SPQ = Schizotypal Personality Questionnaire, AQ = Autism Spectrum Quotient, JART = Japanese Adult Reading Test, IQ = intelligence quotient, WAIS-III = Wechsler Adult Intelligence Scale, Third edition.

**Table S2. Descriptions of exploratory self-report outcomes**

| Domain | Scale | Description | Measure |
| --- | --- | --- | --- |
| Interpersonal reactivity | Interpersonal Reactivity Index (IRI) | The Japanese version of IRI ^[67,68]^ assesses interpersonal reactivity. This self-report questionnaire consists of 28 items rated on 5-point scale, with higher scores indicating stronger interpersonal reactivity. The IRI comprises four subscales: perspective taking, empathic concern, personal distress, and fantasy. Consistent with the original conceptualization, we used subscale scores. | Subscale scores (7–35) |
| Self-consciousness | Self-Consciousness Scale, Revised version (SCSR) | The SCSR ^[69]^ assesses self-consciousness. This self-report questionnaire consists of 22 items rated on a 4-point scale, with higher scores indicating stronger self-consciousness. In this study, we administered only the public self-consciousness and private self-consciousness subscales (16 items), and used the sum of these subscale scores. | Sum of public and private self-consciousness subscale scores (0–48) |
| Social functioning | Social functioning scale (SFS) | The Japanese version of SFS ^[70,71]^ assesses social functioning across seven domains: social withdrawal, interpersonal communication, independence–performance, social activities, independence–competence, recreation, and employment/occupation. Higher scores indicate better social functioning. | Total raw score (0–226) |
| Perceived social support | Multidimensional Scale of Perceived Social Support (MSPSS) | The MSPSS ^[72]^ assesses perceived social support across three sources: family, friends, and a significant other. This self-report questionnaire consists of 12 items rated on a 7-point scale, with higher scores indicating greater perceived social support. | Mean score across all items (1–7) |
| Subjective loneliness | UCLA Loneliness Scale (ULS) | The Japanese version of the ULS ^[73,74]^ assesses subjective feelings of loneliness. This self-report questionnaire consists of 20 items rated on a 4-point scale, with higher scores indicating greater loneliness. | Total raw score (20–80) |
| Self-esteem | Rosenburg Self-Esteem scale (RSE) | The Japanese version of the RSE ^[75,76]^ assesses self-esteem. This self-report questionnaire consists of 10 items rated on a 4-point scale, with higher scores indicating higher self-esteem. | Total raw score (10–40) |

**Table S3. Raw means of outcome measures for SCZ and ASD**

|  | SCZ | | | |  |  | ASD | | | |  |
| --- | --- | --- | --- | --- | --- | --- | --- | --- | --- | --- | --- |
|  | SCST | *n* | CON | *n* | *d* |  | SCST | *n* | CON | *n* | *d* |
| Social cognition: primary outcome | |  |  |  |  |  |  |  |  |  |  |
| FER |  |  |  |  |  |  |  |  |  |  |  |
| Baseline | 16.10 (3.64) | 10 | 15.27 (2.01) | 11 |  |  | 15.79 (2.97) | 14 | 14.67 (2.23) | 12 |  |
| Post | 19.25 (3.15) | 8 | 14.91 (2.63) | 11 | 1.56 |  | 18.23 (3.37) | 13 | 16.44 (3.05) | 9 | 0.26 |
| Follow-up | 19.13 (3.68) | 8 | 14.91 (2.59) | 11 | 1.60 |  | 18.31 (3.86) | 13 | 15.38 (2.39) | 8 | 1.00 |
| FER-BP |  |  |  |  |  |  |  |  |  |  |  |
| Baseline | 14.40 (3.10) | 10 | 12.55 (2.16) | 11 |  |  | 13.08 (3.90) | 13 | 12.58 (2.39) | 12 |  |
| Post | 13.63 (5.10) | 8 | 13.55 (3.05) | 11 | -0.39 |  | 15.92 (3.37) | 12 | 14.56 (1.94) | 9 | 0.75 |
| Follow-up | 15.50 (4.90) | 8 | 13.64 (3.26) | 11 | 0.20 |  | 15.17 (4.57) | 12 | 14.50 (2.20) | 8 | 0.43 |
| RMF |  |  |  |  |  |  |  |  |  |  |  |
| Baseline | 12.90 (3.32) | 10 | 11.91 (2.81) | 11 |  |  | 12.50 (3.06) | 14 | 13.17 (1.59) | 12 |  |
| Post | 10.88 (3.44) | 8 | 13.27 (2.87) | 11 | -2.38 |  | 12.77 (2.74) | 13 | 13.56 (2.19) | 9 | 0.23 |
| Follow-up | 13.00 (2.51) | 8 | 11.82 (2.44) | 11 | 0.53 |  | 12.62 (3.36) | 13 | 14.00 (2.27) | 8 | 0.07 |
| Eyes test |  |  |  |  |  |  |  |  |  |  |  |
| Baseline | 22.60 (2.63) | 10 | 16.09 (5.07) | 11 |  |  | 19.21 (4.21) | 14 | 20.42 (3.53) | 12 |  |
| Post | 20.50 (3.34) | 8 | 16.64 (3.44) | 11 | -0.60 |  | 20.85 (3.48) | 13 | 19.89 (1.90) | 9 | 0.94 |
| Follow-up | 20.63 (4.07) | 8 | 17.55 (4.30) | 11 | -0.89 |  | 20.85 (3.87) | 13 | 20.13 (2.80) | 8 | 0.71 |
| MSCEIT-ME in MCCB | |  |  |  |  |  |  |  |  |  |  |
| Baseline | 82.30 (9.32) | 10 | 79.89 (11.73) | 11 |  |  | 86.99 (8.14) | 14 | 81.78 (6.09) | 12 |  |
| Post | 84.42 (8.75) | 8 | 80.79 (12.75) | 11 | 0.30 |  | 87.43 (10.20) | 13 | 81.36 (5.35) | 9 | 0.12 |
| Follow-up | 81.69 (8.52) | 8 | 80.09 (12.19) | 11 | 0.04 |  | 85.17 (9.81) | 13 | 80.83 (8.63) | 8 | 0.00 |
| Neurocognition |  |  |  |  |  |  |  |  |  |  |  |
| MCCB |  |  |  |  |  |  |  |  |  |  |  |
| Baseline | 279.60 (55.79) | 10 | 267.45 (32.78) | 11 |  |  | 254.64 (65.26) | 14 | 260.83 (32.74) | 12 |  |
| Post | 284.38 (57.04) | 8 | 270.91 (41.80) | 11 | 0.36 |  | 262.62 (66.72) | 13 | 273.33 (32.53) | 9 | 0.05 |
| Follow-up | 285.63 (51.88) | 8 | 280.91 (41.50) | 11 | -0.15 |  | 273.77 (71.29) | 13 | 286.63 (31.02) | 8 | 0.12 |
| Clinical symptoms |  |  |  |  |  |  |  |  |  |  |  |
| PANSS |  |  |  |  |  |  |  |  |  |  |  |
| Baseline | 66.08 (12.04) | 12 | 59.50 (11.17) | 12 |  |  |  |  |  |  |  |
| Post | 65.20 (13.04) | 10 | 59.92 (11.72) | 12 | -0.43 |  |  |  |  |  |  |
| Follow-up | 66.70 (12.58) | 10 | 59.00 (11.11) | 12 | 0.22 |  |  |  |  |  |  |
| CARS2-HF |  |  |  |  |  |  |  |  |  |  |  |
| Baseline |  |  |  |  |  |  | 35.86 (4.74) | 14 | 33.42 (2.57) | 12 |  |
| Post |  |  |  |  |  |  | 32.67 (2.21) | 13 | 34.08 (3.61) | 9 | -0.48 |
| Follow-up |  |  |  |  |  |  | 33.42 (3.30) | 13 | 32.50 (2.68) | 8 | -0.61 |
| SPQ |  |  |  |  |  |  |  |  |  |  |  |
| Baseline | 38.40 (13.88) | 10 | 33.18 (20.11) | 11 |  |  | 42.21 (17.91) | 14 | 37.33 (9.82) | 12 |  |
| Post | 34.75 (16.80) | 8 | 32.91 (19.55) | 11 | -0.55 |  | 42.77 (16.96) | 13 | 35.22 (7.69) | 9 | 0.18 |
| Follow-up | 34.50 (17.55) | 8 | 33.73 (19.27) | 11 | -0.55 |  | 42.85 (17.73) | 13 | 31.50 (6.07) | 8 | 0.61 |
| AQ |  |  |  |  |  |  |  |  |  |  |  |
| Baseline | 27.90 (7.98) | 10 | 25.55 (7.85) | 11 |  |  | 2.56 (0.53) | 14 | 2.54 (0.59) | 12 |  |
| Post | 29.00 (8.69) | 8 | 26.72 (8.20) | 11 | 0.02 |  | 31.31 (7.00) | 13 | 31.22 (6.65) | 9 | 0.33 |
| Follow-up | 27.75 (9.91) | 8 | 27.09 (6.53) | 11 | -0.89 |  | 31.77 (6.18) | 13 | 31.50 (6.70) | 8 | 0.37 |
| Functioning |  |  |  |  |  |  |  |  |  |  |  |
| SOCR |  |  |  |  |  |  |  |  |  |  |  |
| Baseline | 1.30 (1.06) | 10 | 0.91 (0.70) | 11 |  |  | 1.07 (0.83) | 14 | 1.25 (1.06) | 12 |  |
| Post | 1.13 (1.13) | 8 | 0.82 (0.87) | 11 | 0.22 |  | 1.23 (0.73) | 13 | 1.44 (1.13) | 9 | 0.20 |
| Follow-up | 1.56 (1.01) | 8 | 0.63 (0.92) | 11 | 0.46 |  | 1.17 (0.94) | 13 | 1.00 (0.89) | 8 | 1.06 |
| WHOQOL26 |  |  |  |  |  |  |  |  |  |  |  |
| Baseline | 2.91 (0.54) | 10 | 3.04 (0.65) | 11 |  |  | 2.56 (0.53) | 14 | 2.54 (0.59) | 12 |  |
| Post | 2.90 (0.65) | 8 | 3.10 (0.59) | 11 | -0.42 |  | 2.61 (0.59) | 13 | 2.76 (0.58) | 9 | 0.08 |
| Follow-up | 5.50 (2.20) | 8 | 5.91 (2.12) | 11 | 0.16 |  | 5.77 (1.48) | 13 | 5.25 (1.16) | 8 | 0.69 |
| GAF |  |  |  |  |  |  |  |  |  |  |  |
| Baseline | 49.50 (8.78) | 10 | 46.73 (6.08) | 11 |  |  | 47.93 (8.71) | 14 | 49.92 (8.73) | 12 |  |
| Post | 48.88 (10.26) | 8 | 46.73 (6.65) | 11 | 0.22 |  | 49.46 (8.42) | 13 | 52.00 (7.78) | 9 | 0.87 |
| Follow-up | 49.11 (7.90) | 8 | 46.25 (6.82) | 11 | -0.43 |  | 50.58 (9.19) | 13 | 49.91 (6.49) | 8 | 0.98 |

Note. SCZ = schizophrenia, ASD = autism spectrum disorder (the ASD category includes 3 participants with comorbid SCZ), SCST = social cognitive skills training group, CON = control group, post = post intervention, FER = Facial Emotion Recognition task, FER-BP = Facial Emotion Recognition through Brief Presentation task, RMF = Reading the Mind in Films test, Eyes test = Reading the mind in the Eyes test, MSCEIT-ME = Managing Emotions of the Mayer-Salovey-Caruso Emotional Intelligence Test, MCCB = MATRICS Consensus Cognitive Battery, PANSS = Positive and Negative Syndrome Scale, CARS2-HF = Childhood Autism Rating Sale, Second edition, High Functioning version, SPQ = Schizotypal Personality Questionnaire, AQ = Autism Spectrum Quotient, SOCR = Social Outcome Composite Rating, WHOQOL-26 = World Health Organization Quality of Life-26, GAF = Global Assessment of Functioning.

**Table S4. Exploratory self-reported outcomes:** **Estimated marginal means across assessments**

|  | SCST  Mean (SE) | CON  Mean (SE) | Group | Time | G.×T.  Interaction | G.×T.×D.  Interaction |
| --- | --- | --- | --- | --- | --- | --- |
| Functioning |  |  |  |  |  |  |
| IRI perspective taking |  |  |  |  |  |  |
| Baseline | 20.24  (0.99) | 20.14  (1.00) | *F*(1,77.70) =0.41  *p* = .524  *f*^2^ < 0.01 | *F*(1,36.93) = 1.75  *p* = .195  *f*^2^ = 0.05 | *F*(1,36.93) = 1.34  *p* = .255  *f*^2^ = 0.04 | *F*(1,36.93) = 1.61  *p* = .213  *f*^2^ = 0.04 |
| Follow-up | 20.32  (1.02) | 21.36  (1.04) |  |  |  |  |
| IRI empathic concern |  |  |  |  |  |  |
| Baseline | 24.03  (0.90) | 25.13  (0.91) | *F*(1,76.53) = 1.32  *p* = .253  *f*^2^ = 0.02 | *F*(1,37.59) = 0.03  *p* = .869  *f*^2^ < 0.01 | *F*(1,37.59) = 1.08  *p* = .305  *f*^2^ = 0.03 | *F*(1,37.59) = 0.33  *p* = .571  *f*^2^ < 0.01 |
| Follow-up | 24.60  (0.93) | 24.72  (0.95) |  |  |  |  |
| IRI personal distress |  |  |  |  |  |  |
| Baseline | 25.99  (1.07) | 26.70  (1.08) | *F*(1,78.95) = 1.42  *p* = .237  *f*^2^ = 0.02 | *F*(1,37.54) = 0.35  *p* = .556  *f*^2^ < 0.01 | *F*(1,37.54) = 3.15  *p* = .084  *f*^2^ = 0.08 | *F*(1,37.54) = 0.08  *p* = .780  *f*^2^ < 0.01 |
| Follow-up | 27.09  (1.10) | 26.15  (1.12) |  |  |  |  |
| IRI fantasy |  |  |  |  |  |  |
| Baseline | 23.48  (1.33) | 22.51  (1.35) | *F*(1,77.94) = 0.86  *p* = 0.36  *f*^2^ = 0.01 | *F*(1,36.90) = 1.00  *p* = .323  *f*^2^ = 0.03 | *F*(1,36.90) = 1.20  *p* = .281  *f*^2^ = 0.03 | *F*(1,36.90) = 1.48  *p* = .231  *f*^2^ = 0.04 |
| Follow-up | 22.10  (1.38) | 22.57  (1.40) |  |  |  |  |
| SCSR |  |  |  |  |  |  |
| Baseline | 24.80  (1.90) | 27.20  (1.91) | *F*(1,78.48) = 1.47  *p* = .229  *f*^2^ = 0.02 | *F*(1,37.70) = 0.52  *p* = .475  *f*^2^ = 0.01 | *F*(1,37.70) = 1.14  *p* = .293  *f*^2^ = 0.03 | *F*(1,37.70) = 0.36  *p* = .553  *f*^2^ < 0.01 |
| Follow-up | 25.10  (1.96) | 25.60  (1.96) |  |  |  |  |
| SFS |  |  |  |  |  |  |
| Baseline | 112.81  (4.99) | 117.18  (5.03) | *F*(1,66.13) = 0.08  *p* = .776  *f*^2^ < 0.01 | *F*(1,40.00) = 2.93  *p* = .095  *f*^2^ < 0.07 | *F*(1,40.00) = 0.02  *p* = .887  *f*^2^ < 0.01 | *F*(1,40.00) = 1.95  *p* = .170  *f*^2^ = 0.05 |
| Follow-up | 106.43  (5.15) | 111.78  (5.43) |  |  |  |  |
| MSPSS |  |  |  |  |  |  |
| Baseline | 3.58  (0.28) | 4.01  (0.28) | *F*(1,72.16) = 1.03  *p* = .313  *f*^2^ = 0.01 | *F*(1,38.71) = 0.11  *p* = .747  *f*^2^ < 0.01 | *F*(1,38.71) = 0.32  *p* = .576  *f*^2^ < 0.01 | *F*(1,38.71) = 0.99  *p* = .325  *f*^2^ = 0.03 |
| Follow-up | 3.73  (0.29) | 3.97  (0.28) |  |  |  |  |
| ULS |  |  |  |  |  |  |
| Baseline | 54.91  (2.00) | 52.97  (2.01) | *F*(1,76.22) = 0.15  *p* = .702  *f*^2^ < 0.01 | *F*(1,37.83) = 1.52  *p* = .225  *f*^2^ = 0.04 | *F*(1,37.83) = 0.03  *p* = .854  *f*^2^ < 0.01 | *F*(1,37.83) = 0.65  *p* = .427  *f*^2^ = 0.02 |
| Follow-up | 53.80  (2.07) | 51.47  (2.11) |  |  |  |  |
| RSE |  |  |  |  |  |  |
| Baseline | 24.21  (1.61) | 25.14  (1.62) | *F*(1,76.20) < 0.01  *p* = .966  *f*^2^ < 0.01 | *F*(1,38.22) = 1.92  *p* = .174  *f*^2^ = 0.05 | *F*(1,38.22) = 0.21  *p* = .647  *f*^2^ < 0.01 | *F*(1,38.22) = 0.44  *p* = .509  *f*^2^ = 0.01 |
| Follow-up | 25.00  (1.67) | 26.72  (1.71) |  |  |  |  |

Note. n = 47. SCST = social cognitive skills training, CON = control, SE = standard error, G.×T. = group by time, G.×T.×D. = group by time by diagnosis, IRI = Interpersonal Reactivity Index, SCSR = Self-Consciousness Scale, Revised version, SFS = Social functioning scale, MSPSS = Multidimensional Scale of Perceived Social Support, ULS = UCLA Loneliness Scale, RSE = Rosenburg Self-Esteem scale.

**Table S5. Baseline comparison between SCZ and ASD**

|  | SCZ (n = 21)  Mean (SD) | ASD (n = 23)  Mean (SD) |  |
| --- | --- | --- | --- |
| Age (years) | 47.24 (9.37) | 35.13 (11.37) | *t*(42) = 3.83, *p* < .001 |
| Sex (Male%) | 33.33 | 73.91 | *χ*^2^(1)= 5.75, *p* = .017 |
| Education (y) | 14.75 (2.59) | 13.83 (2.15) | *t*(42) = -0.85, *p* = .399 |
| SPQ | 35.67 (17.21) | 40.09 (15.39) | *t*(42) = -0.90, *p* = .375 |
| AQ | 26.67 (7.81) | 31.74 (6.14) | *t*(42) = -2.41, *p* = .022 |
| JART-estimated-IQ | 108.71 (9.61) | 108.48 (7.75) | *t*(42) = 0.09, *p* = .930 |
| WAIS-III-estimated-IQ | 94.48 (18.30) | 97.30 (22.22) | *t*(42) = -0.46, *p* = .650 |
| Digit Symbol-Coding | 68.29 (19.54) | 73.70 (24.70) | *t*(42) = -0.80, *p* = .429 |
| Matrix Reasoning | 16.19 (5.92) | 18.78 (5.16) | *t*(42) = -1.55, *p* = .129 |
| Digit Span | 16.38 (5.05) | 18.61 (4.45) | *t*(42) = -1.55, *p* = .128 |
| Information | 17.67 (4.58) | 17.61 (4.18) | *t*(42) = 0.04, *p* = .930 |
| FER | 15.67 (2.85) | 15.48 (2.69) | *t*(42) = 0.23, *p* = .824 |
| FER-BP | 13.43 (2.75) | 12.82 (3.42) | *t*(41) = 0.64, *p* = .525 |
| RMF | 12.38 (3.02) | 12.96 (2.36) | *t*(42) = -0.71, *p* = .485 |
| Eyes test | 19.19 (5.20) | 19.65 (4.05) | *t*(42) = -0.33, *p* = .744 |
| MCCB | 304.57 (51.86) | 295.30 (57.30) | *t*(42) = -0.56, *p* = .579 |
| MSCEIT-ME | 81.03 (10.46) | 83.82 (7.71) | *t*(42) = -1.01, *p* = .319 |
| Trail Making Test-A | 37.24 (18.04) | 40.67 (19.41) | *t*(42) = -0.61, *p* = .548 |
| BACS Symbol Coding | 52.33 (12.46) | 53.43 (15.08) | *t*(42) = -0.26, *p* = .795 |
| HVLT-R | 23.38 (5.42) | 25.52 (6.33) | *t*(42) = -1.20, *p* = .238 |
| WMS-III Spatial Span | 16.33 (2.78) | 16.57 (4.18) | *t*(42) = -0.21, *p* = .832 |
| Letter-Number Span | 11.14 (3.50) | 12.87 (2.47) | *t*(42) = -1.90, *p* = .065 |
| NAB Mazes | 19.29 (5.10) | 18.39 (7.73) | *t*(42) = 0.45, *p* = .657 |
| BVMT-R | 21.76 (6.66) | 20.43 (9.28) | *t*(42) = 0.54, *p* = .593 |
| Category Fluency | 20.62 (6.68) | 17.87 (4.48) | *t*(42) = 1.62, *p* = .114 |
| CPT-IP | 2.64 (0.61) | 2.82 (0.83) | *t*(42) = -0.83, *p* = .414 |
| SOCR | 1.10 (0.89) | 1.22 (0.95) | *t*(42) = -0.44, *p* = .664 |
| WHOQOL26 | 2.98 (0.59) | 2.57 (0.56) | *t*(42) = 2.36, *p* = .024 |
| GAF | 48.05 (7.43) | 49.04 (8.95) | *t*(42) = -0.40, *p* = .693 |

Note. SCZ = schizophrenia, ASD = autism spectrum disorder, SD = standard deviation, SPQ = Schizotypal Personality Questionnaire, AQ = Autism Spectrum Quotient, JART = Japanese Adult Reading Test, IQ = intelligence quotient, WAIS-III = Wechsler Adult Intelligence Scale, Third edition, FER = Facial Emotion Recognition task, FER-BP = Facial Emotion Recognition through Brief Presentation task, RMF = Reading the Mind in Films test, Eyes test = Reading the Mind in the Eyes test, MCCB = MATRICS Consensus Cognitive Battery, MSCEIT-ME = Managing Emotions subtest of the Mayer-Salovey-Caruso Emotional Intelligence Test, BACS = Brief Assessment of Cognition in Schizophrenia, HVLT-R = Hopkins Verbal Learning Test, Revised, WMS-III = Wechsler Memory Scale, Third Edition, NAB = Neuropsychological Assessment Battery, BVMT-R = Brief Visuospatial Memory Test, Revised, CPT-IP = Continuous Performance Test, Identical Pairs, SOCR = Social Outcome Composite Rating, WHOQOL-26 = World Health Organization Quality of Life-26, GAF = Global Assessment of Functioning.

**Table S6. Baseline Pearson correlations between neurocognition and social cognition in SCZ**

|  | FER | FER-BP | RMF | Eyes test | MSCEIT-ME |
| --- | --- | --- | --- | --- | --- |
| WAIS-III-estimated IQ | *r* = 0.65  *t*(19) = 3.76  *p* = .001 | *r* = 0.63  *t*(19) = 3.55  *p* = .002 | *r* = 0.60  *t*(19) = 3.25  *p* = .004 | *r* = 0.21  *t*(19) = 0.92  *p* = .368 | *r* = 0.40  *t*(19) = 1.93  *p* = .069 |
| Digit Symbol-Coding | *r* = 0.35  *t*(19) = 1.61  *p* = .123 | *r* = 0.48  *t*(19) = 2.38  *p* = .028 | *r* = 0.54  *t*(19) = 2.79  *p* = .012 | *r* = 0.22  *t*(19) = 0.97  *p* = .345 | *r* = 0.54  *t*(19) = 2.80  *p* = .011 |
| Matrix Reasoning | *r* = 0.71  *t*(19) = 4.41  *p* < .001 | *r* = 0.67  *t*(19) = 3.91  *p* < .001 | *r* = 0.65  *t*(19) = 3.75  *p* = .001 | *r* = 0.33  *t*(19) = 1.55  *p* = .138 | *r* = 0.61  *t*(19) = 3.40  *p* = .003 |
| Digit Span | *r* = 0.69  *t*(19) = 3.92  *p* < .001 | *r* = 0.55  *t*(19) = 2.86  *p* = .010 | *r* = 0.60  *t*(19) = 3.31  *p* = .004 | *r* = 0.40  *t*(19) = 1.90  *p* = .072 | *r* = 0.20  *t*(19) = 0.87  *p* = .395 |
| Information | *r* = 0.10  *t*(19) = 0.45  *p* = .659 | *r* = 0.14  *t*(19) = 0.61  *p* = .548 | *r* = 0.26  *t*(19) = 0.96  *p* = .348 | *r* = -0.08  *t*(19) = -0.35  *p* = .733 | *r* = 0.30  *t*(19) = 1.39  *p* = .193 |
| Neurocognition total | *r* = 0.68  *t*(19) = 4.04  *p* < .001 | *r* = 0.69  *t*(19) = 4.18  *p* < .001 | *r* = 0.62  *t*(19) = 3.46  *p* = .003 | *r* = 0.44  *t*(19) = 2.12  *p* = .047 | *r* = 0.60  *t*(19) = 3.27  *p* = .004 |
| Trail Making Test-A | *r* = -0.65  *t*(19) = -3.75  *p* = .001 | *r* = -0.61  *t*(19) = -3.34  *p* = .003 | *r* = -0.57  *t*(19) = -3.06  *p* = .006 | *r* = -0.42  *t*(19) = -2.02  *p* = .057 | *r* = -0.34  *t*(19) = -1.59  *p* = .129 |
| BACS Symbol Coding | *r* = 0.34  *t*(19) = 1.56  *p* = .136 | *r* = 0.43  *t*(19) = 2.05  *p* = .054 | *r* = 0.52  *t*(19) = 2.62  *p* = .017 | *r* = 0.29  *t*(19) = 1.31  *p* = .205 | *r* = 0.62  *t*(19) = 3.41  *p* = .003 |
| HVLT-R | *r* = 0.42  *t*(19) = 2.03  *p* = .056 | *r* = 0.58  *t*(19) = 3.07  *p* = .006 | *r* = 0.65  *t*(19) = 3.76  *p* = .001 | *r* = 0.40  *t*(19) = 1.93  *p* = .070 | *r* = 0.65  *t*(19) = 3.75  *p* = .002 |
| WMS-III Spatial Span | *r* = 0.32  *t*(19) = 1.46  *p* = .161 | *r* = 0.31  *t*(19) = 1.44  *p* = .166 | *r* = 0.23  *t*(19) = 1.05  *p* = .308 | *r* = -0.01  *t*(19) = -0.03  *p* = .972 | *r* = 0.36  *t*(19) = 1.67  *p* = .110 |
| Letter-Number Span | *r* = 0.56  *t*(19) = 2.96  *p* = .008 | *r* = 0.48  *t*(19) = 2.37  *p* = .029 | *r* = 0.63  *t*(19) = 3.56  *p* = .002 | *r* = 0.42  *t*(19) = 2.03  *p* = .057 | *r* = 0.24  *t*(19) = 1.08  *p* = .193 |
| NAB Mazes | *r* = 0.53  *t*(19) = 2.72  *p* = .009 | *r* = 0.43  *t*(19) = 2.09  *p* = .049 | *r* = 0.34  *t*(19) = 1.56  *p* = .136 | *r* = 0.05  *t*(19) = 0.24  *p* = .815 | *r* = 0.53  *t*(19) = 2.71  *p* = .014 |
| BVMT-R | *r* = 0.26  *t*(19) = 1.15  *p* = .264 | *r* = 0.58  *t*(19) = 3.08  *p* = .006 | *r* = 0.46  *t*(19) = 2.25  *p* = .036 | *r* = 0.50  *t*(19) = 2.51  *p* = .021 | *r* = 0.52  *t*(19) = 2.66  *p* = .015 |
| Category Fluency | *r* = 0.34  *t*(19) = 1.56  *p* = .136 | *r* = 0.16  *t*(19) = 0.73  *p* = .476 | *r* = 0.24  *t*(19) = 1.07  *p* = .300 | *r* = 0.28  *t*(19) = 1.26  *p* = .222 | *r* = 0.38  *t*(19) = 1.80  *p* = .088 |
| CPT-IP | *r* = -0.31  *t*(19) = 1.41  *p* = .175 | *r* = -0.34  *t*(19) = 1.56  *p* = .136 | *r* = 0.41  *t*(19) = 1.98  *p* = .062 | *r* = 0.17  *t*(19) = 0.74  *p* = .468 | *r* = 0.39  *t*(19) = 1.85  *p* = .080 |

Note. *n* = 21. SCZ = schizophrenia, FER = Facial Emotion Recognition task, FER-BP = Facial Emotion Recognition through Brief Presentation task, RMF = Reading the Mind in Films test, Eyes test = Reading the Mind in the Eyes test, MSCEIT-ME = Managing Emotions subtest of the Mayer-Salovey-Caruso Emotional Intelligence Test, WAIS-III = Wechsler Adult Intelligence Scale, Third edition, IQ = intelligence quotient, BACS = Brief Assessment of Cognition in Schizophrenia, HVLT-R = Hopkins Verbal Learning Test, Revised, WMS-III = Wechsler Memory Scale, Third Edition, NAB = Neuropsychological Assessment Battery, BVMT-R = Brief Visuospatial Memory Test, Revised, CPT-IP = Continuous Performance Test, Identical Pairs.

**Table S7. Baseline Pearson correlations between neurocognition and social cognition in ASD**

|  | FER | FER-BP | RMF | Eyes test | MSCEIT-ME |
| --- | --- | --- | --- | --- | --- |
| WAIS-III-estimated IQ | *r* = 0.12  *t*(21) = 0.57  *p* = .577 | *r* = 0.45  *t*(20) = 2.24  *p* = .036 | *r* = 0.76  *t*(21) = 5.32  *p* < .001 | *r* = 0.51  *t*(21) = 2.68  *p* = .014 | *r* = 0.50  *t*(21) = 2.65  *p* = .015 |
| Digit Symbol-Coding | *r* = 0.03  *t*(21) = 0.15  *p* = .879 | *r* = 0.44  *t*(20) = 2.18  *p* = .041 | *r* = 0.36  *t*(21) = 1.78  *p* = .090 | *r* = 0.28  *t*(21) = 1.35  *p* = .190 | *r* = 0.16  *t*(21) = 0.74  *p* = .469 |
| Matrix Reasoning | *r* = 0.21  *t*(21) = 1.00  *p* = .327 | *r* = 0.71  *t*(20) = 4.53  *p* < .001 | *r* = 0.54  *t*(21) = 2.96  *p* = .007 | *r* = 0.39  *t*(21) = 1.91  *p* = .069 | *r* = -0.03  *t*(21) = -0.13  *p* = .901 |
| Digit Span | *r* = 0.31  *t*(21) = 1.49  *p* = .152 | *r* = 0.18  *t*(20) = 0.84  *p* = .412 | *r* = 0.66  *t*(21) = 4.07  *p* < .001 | *r* = 0.53  *t*(21) = 2.90  *p* = .009 | *r* = 0.37  *t*(21) = 1.81  *p* = .085 |
| Information | *r* = 0.10  *t*(21) = 0.47  *p* = .643 | *r* = -0.11  *t*(20) = -0.48  *p* = .640 | *r* = 0.40  *t*(21) = 1.99  *p* = .060 | *r* = 0.28  *t*(21) = 1.33  *p* = .197 | *r* = 0.62  *t*(21) = 3.62  *p* = .002 |
| Neurocognition total | *r* = 0.01  *t*(21) = 0.05  *p* = .962 | *r* = 0.58  *t*(20) = 3.19  *p* = .005 | *r* = 0.68  *t*(21) = 4.27  *p* < .001 | *r* = 0.53  *t*(21) = 2.83  *p* = .010 | *r* = 0.35  *t*(21) = 1.70  *p* = .103 |
| Trail Making Test-A | *r* = 0.12  *t*(21) = 0.54  *p* = .592 | *r* = -0.57  *t*(20) = -3.06  *p* = .006 | *r* = -0.56  *t*(21) = -3.09  *p* = .006 | *r* = -0.43  *t*(21) = -2.16  *p* = .043 | *r* = 0.04  *t*(21) = 0.17  *p* = .868 |
| BACS Symbol Coding | *r* = 0.13  *t*(21) = 0.58  *p* = .568 | *r* = 0.56  *t*(20) = 3.04  *p* = .006 | *r* = 0.52  *t*(21) = 2.77  *p* = .012 | *r* = 0.52  *t*(21) = 2.79  *p* = .011 | *r* = 0.24  *t*(21) = 1.15  *p* = .263 |
| HVLT-R | *r* = 0.01  *t*(21) = 0.05  *p* = .959 | *r* = 0.40  *t*(20) = 1.98  *p* = .062 | *r* = 0.62  *t*(21) = 3.63  *p* = .002 | *r* = 0.66  *t*(21) = 4.04  *p* = .001 | *r* = 0.22  *t*(21) = 1.02  *p* = .317 |
| WMS-III Spatial Span | *r* = 0.18  *t*(21) = 0.82  *p* = .420 | *r* = 0.52  *t*(20) = 2.70  *p* = .014 | *r* = 0.40  *t*(21) = 1.99  *p* = .060 | *r* = 0.63  *t*(21) = 3.71  *p* = .001 | *r* = 0.24  *t*(21) = 1.14  *p* = .268 |
| Letter-Number Span | *r* = 0.66  *t*(21) = 4.00  *p* = .001 | *r* = 0.39  *t*(20) = 1.89  *p* = .073 | *r* = 0.42  *t*(21) = 2.11  *p* = .047 | *r* = 0.49  *t*(21) = 2.60  *p* = .017 | *r* = 0.10  *t*(21) = 0.46  *p* = .652 |
| NAB Mazes | *r* = 0.14  *t*(21) = 0.65  *p* = .527 | *r* = 0.45  *t*(20) = 2.27  *p* = .034 | *r* = 0.53  *t*(21) = 2.83  *p* = .010 | *r* = 0.21  *t*(21) = 0.98  *p* = .338 | *r* = 0.10  *t*(21) = 0.48  *p* = .638 |
| BVMT-R | *r* = 0.07  *t*(21) = 0.33  *p* = .747 | *r* = 0.51  *t*(20) = 2.66  *p* = .015 | *r* = 0.21  *t*(21) = 0.99  *p* = .331 | *r* = 0.19  *t*(21) = 0.91  *p* = .375 | *r* = -0.09  *t*(21) = -0.44  *p* = .666 |
| Category Fluency | *r* = -0.01  *t*(21) = -0.03  *p* = .979 | *r* = 0.28  *t*(20) = 1.32  *p* = .202 | *r* = 0.47  *t*(21) = 2.45  *p* = .023 | *r* = 0.44  *t*(21) = 2.25  *p* = .035 | *r* = 0.48  *t*(21) = 2.51  *p* = .020 |
| CPT-IP | *r* = 0.18  *t*(21) = 0.82  *p* = .421 | *r* = 0.26  *t*(20) = 1.21  *p* = .240 | *r* = 0.52  *t*(21) = 2.81  *p* = .010 | *r* = 0.38  *t*(21) = 1.89  *p* = .072 | *r* = 0.30  *t*(21) = 1.42  *p* = .169 |

Note. *n* = 23. ASD = autism spectrum disorder, FER = Facial Emotion Recognition task, FER-BP = Facial Emotion Recognition through Brief Presentation task, RMF = Reading the Mind in Films test, Eyes test = Reading the Mind in the Eyes test, MSCEIT-ME = Managing Emotions subtest of the Mayer-Salovey-Caruso Emotional Intelligence Test, WAIS-III = Wechsler Adult Intelligence Scale, Third edition, IQ = intelligence quotient, BACS = Brief Assessment of Cognition in Schizophrenia, HVLT-R = Hopkins Verbal Learning Test, Revised, WMS-III = Wechsler Memory Scale, Third Edition, NAB = Neuropsychological Assessment Battery, BVMT-R = Brief Visuospatial Memory Test, Revised, CPT-IP = Continuous Performance Test, Identical Pairs.

**Table S8. Sensitivity analysis excluding participants with comorbid SCZ and ASD: Estimated marginal means across assessments**

|  | SCST  Mean (SE) | CON  Mean (SE) | Group | Time | G.×T.  Interaction | G.×T.×D.  Interaction |
| --- | --- | --- | --- | --- | --- | --- |
| Social cognition |  |  |  |  |  |  |
| Eyes test |  |  |  |  |  |  |
| Baseline to Post-intervention | |  |  |  |  |  |
| Baseline | 20.88  (0.79) | 18.14  (0.79) | *F*(1,59.44) = 2.80  *p* = .100  *f*^2^ = 0.05 | *F*(1,36.76) = 0.05  *p* = .822  *f*^2^ < 0.01 | *F*(1,36.76) = 0.15  *p* = .704  *f*^2^ < 0.01 | *F*(1,36.76) = 3.96  *p* = .054  *f*^2^ = 0.11 |
| Post-intervention | 20.54  (0.84) | 18.22  (0.84) |  |  |  |  |
| Baseline to Follow-up | |  |  |  |  |  |
| Baseline | 20.88  (0.85) | 18.14  (0.85) | *F*(1,64.10) = 3.94  *p* = .052  *f*^2^ = 0.06 | *F*(1,35.94) = 0.13  *p* = .722  *f*^2^ < 0.01 | *F*(1,35.94) = 0.88  *p* = .355  *f*^2^ = 0.02 | *F*(1,35.94) = 5.13  *p* = .030  *f*^2^ = 0.14 |
| Follow-up | 20.57  (0.90) | 18.83  (0.92) |  |  |  |  |
|  |  |  |  |  |  |  |
| Functioning |  |  |  |  |  |  |
| GAF |  |  |  |  |  |  |
| Baseline to Follow-up | |  |  |  |  |  |
| Baseline | 48.87  (1.74) | 48.32  (1.73) | *F*(1,66.31) = 0.13  *p* = .718  *f*^2^ < 0.01 | *F*(1,33.56) = 0.34  *p* = .564  *f*^2^ = 0.01 | *F*(1,33.56) = 2.09  *p* = .157  *f*^2^ = 0.06 | *F*(1,33.56) = 5.56  *p* = .024  *f*^2^ = 0.17 |
| Follow-up | 49.99  (1.76) | 47.84  (1.77) |  |  |  |  |

Note. *n* = 44. SCZ = schizophrenia, ASD = autism spectrum disorder, SCST = social cognitive skills training, CON = control, SE = standard error, G.×T. = group by time, G.×T.×D. = group by time by diagnosis, Eyes test = Reading the Mind in the Eyes test, GAF = Global Assessment of Functioning.

**Table S9. Baseline characteristics of participants with comorbid SCZ and ASD relative to participants with SCZ only and ASD only**

|  | SCZ+ASD (*n* = 3)  Mean (SD) | SCZ (n = 21)  Mean (SD) | ASD (n = 23)  Mean (SD) |
| --- | --- | --- | --- |
| Age (years) | 37.00 (4.00) | 47.24 (9.37) | 35.13 (11.37) |
| Education (y) | 13.67 (4.04) | 14.75 (2.59) | 13.83 (2.15) |
| Duration (y) | 14.67 (4.99) | 20.95 (9.99) | NA |
| Sex (Male%) | 66.67 | 33.33 | 73.91 |
| Dosage (mg/d, RPD eq.) | 2.03 (0.45) | 4.72 (3.15) | 1.23 (2.07) |
| PANSS Positive | 13.33 (0.58) | 14.48 (3.67) | NA |
| PANSS Negative | 15.67 (2.31) | 17.67 (4.45) | NA |
| CARS2-HF | 34.00 (1.80) | NA | 34.84 (4.24) |
| SPQ | 39.00 (9.17) | 35.67 (17.21) | 40.09 (15.39) |
| AQ | 31.67 (2.89) | 26.67 (7.81) | 31.74 (6.14) |
| JART-estimated-IQ | 105.33 (5.69) | 108.71 (9.61) | 108.48 (7.75) |
| WAIS-III-estimated-IQ | 92.67 (20.03) | 94.48 (18.30) | 97.30 (22.22) |
| FER | 13.67 (2.08) | 15.67 (2.85) | 15.48 (2.69) |
| FER-BP | 13.00 (1.00) | 13.43 (2.75) | 12.82 (3.42) |
| RMF | 11.67 (3.51) | 12.38 (3.02) | 12.96 (2.36) |
| Eyes test | 20.67 (2.52) | 19.19 (5.20) | 19.65 (4.05) |
| MSCEIT-ME | 90.50 (3.13) | 81.03 (10.46) | 83.82 (7.71) |
| MCCB | 283.67 (41.30) | 304.57 (51.86) | 295.30 (57.30) |
| SOCR | 0.67 (0.58) | 1.10 (0.89) | 1.22 (0.95) |
| WHOQOL26 | 2.39 (0.49) | 2.98 (0.59) | 2.57 (0.56) |
| GAF | 47.33 (6.43) | 48.05 (7.43) | 49.04 (8.95) |

Note. SCZ = schizophrenia, ASD = autism spectrum disorder, SD = standard deviation, RPD eq. = risperidone equivalence, PANSS = Positive and Negative Syndrome Scale, CARS2-HF = Childhood Autism Rating Scale, Second edition, High Functioning version, SPQ = Schizotypal Personality Questionnaire, AQ = Autism Spectrum Quotient, JART = Japanese Adult Reading Test, IQ = intelligence quotient, WAIS-III = Wechsler Adult Intelligence Scale, Third edition, FER = Facial Emotion Recognition task, FER-BP = Facial Emotion Recognition through Brief Presentation task, RMF = Reading the Mind in Films test, Eyes test = Reading the Mind in the Eyes test, MSCEIT-ME = Managing Emotions subtest of the Mayer-Salovey-Caruso Emotional Intelligence Test, MCCB = MATRICS Consensus Cognitive Battery, SOCR = Social Outcome Composite Rating, WHOQOL-26 = World Health Organization Quality of Life-26, GAF = Global Assessment of Functioning.

**References**

67. Davis MH. A multidimensional approach to individual differences in empathy. JSAS Cat Select Doc Psychol. 1980;10:85.
68. Himichi T, Osanai H, Goto T, Fujita H, Kawamura Y, Davis MH, Nomura M. Development of a Japanese version of the Interpersonal Reactivity Index. Jpn J Psychol. 2017;88:61–71.
69. Scheier MF, Carver CS. The Self-Consciousness Scale: A revised version for use with general populations. J Appl Soc Psychol. 1985;15:687–699.
70. Birchwood M, Smith J, Cochrane R, Wetton S, Copestake S. The Social Functioning Scale: The development and validation of a new scale of social adjustment for use in family intervention programmes with schizophrenic patients. Br J Psychiatry. 1990;157:853–859.
71. Nemoto T, Fujii C, Miura U, Chino B, Kobayashi H, Yamazawa R, Murakami M, Kashima H, Mizuno M. Development of the Japanese version of the Social Functioning Scale (SFS) and examination of its reliability and validity. Jpn Bull Soc Psychiatry. 2008;17:188–195.
72. Zimet GD, Dahlem NW, Zimet SG, Farley GK. The Multidimensional Scale of Perceived Social Support. J Pers Assess. 1988;52:30–41.
73. Russell DW. UCLA Loneliness Scale (Version 3): Reliability, validity, and factor structure. J Pers Assess. 1996;66:20–40.
74. Masuda Y, Tadaka E, Dai Y. Reliability and validity of the Japanese version of the UCLA loneliness scale version 3 among the older population. J Jpn Acad Community Health Nurs. 2012;15:25–32.
75. Rosenberg M. Society and the Adolescent Self-Image. Princeton, NJ: Princeton University Press; 1965.
76. Yamamoto M, Matsui Y, Yamanari Y. The structure of perceived aspects of self. Jpn J Educ Psychol. 1982;30:64–68.
